# Versatile Macrocyclic Platform for the Complexation of [^nat^Y/^90^Y]Yttrium and Lanthanide Ions

**DOI:** 10.1021/acs.inorgchem.2c00378

**Published:** 2022-04-14

**Authors:** Charlene Harriswangler, Laura Caneda-Martínez, Olivier Rousseaux, David Esteban-Gómez, Olivier Fougère, Rosa Pujales-Paradela, Laura Valencia, M. Isabel Fernández, Nicolas Lepareur, Carlos Platas-Iglesias

**Affiliations:** † Centro de Investigacións Científicas Avanzadas (CICA) and Departamento de Química, Facultade de Ciencias, Universidade da Coruña, 15071 Galicia, A Coruña, Spain; ‡ Groupe Guerbet, Centre de Recherche d’Aulnay-sous-Bois, BP 57400, 95943 Roissy CdG Cedex, France; § Departamento de Química Inorgánica, Facultad de Ciencias, Universidade de Vigo, As Lagoas, Marcosende, 36310 Pontevedra, Spain; ∥ Univ Rennes, Centre Eugène Marquis, Inrae, Inserm, Institut NUMECAN (Nutrition, Métabolismes et Cancer)—UMR_A 1341, UMR_S 1241, F-35000 Rennes, France

## Abstract

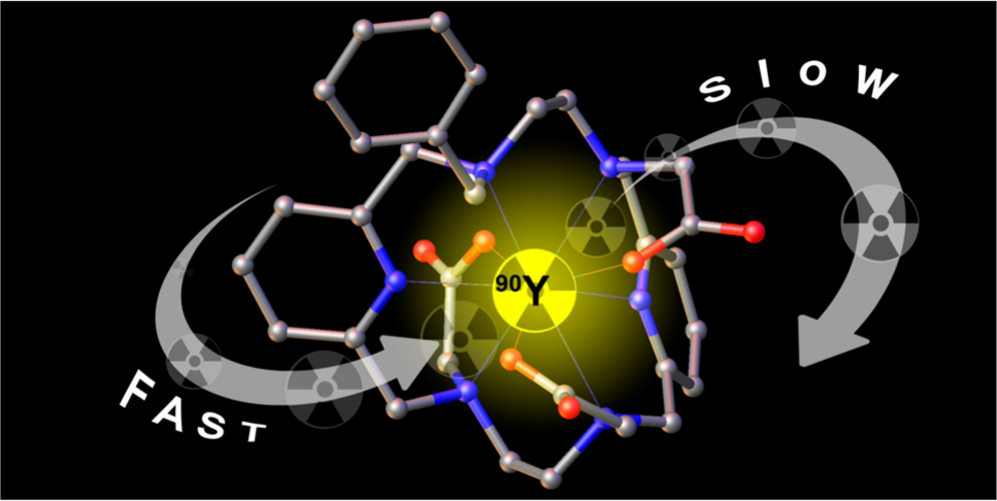

We report a macrocyclic ligand (H_3_**L**^**6**^) based on a 3,6,10,13-tetraaza-1,8(2,6)-dipyridinacyclotetradecaphane platform containing three acetate pendant arms and a benzyl group attached to the fourth nitrogen atom of the macrocycle. The X-ray structures of the Y**L**^**6**^ and Tb**L**^**6**^ complexes reveal nine coordination of the ligand to the metal ions through the six nitrogen atoms of the macrocycle and three oxygen atoms of the carboxylate pendants. A combination of NMR spectroscopic studies (^1^H, ^13^C, and ^89^Y) and DFT calculations indicated that the structure of the Y**L**^**6**^ complex in the solid state is maintained in an aqueous solution. The detailed study of the emission spectra of the Eu**L**^**6**^ and Tb**L**^**6**^ complexes revealed Ln^3+^-centered emission with quantum yields of 7.0 and 60%, respectively. Emission lifetime measurements indicate that the ligand offers good protection of the metal ions from surrounding water molecules, preventing the coordination of water molecules. The Y**L**^**6**^ complex is remarkably inert with respect to complex dissociation, with a lifetime of 1.7 h in 1 M HCl. On the other hand, complex formation is fast (∼1 min at pH 5.4, 2 × 10^–5^ M). Studies using the ^90^Y-nuclide confirmed fast radiolabeling since [^90^Y]Y**L**^**6**^ is nearly quantitatively formed (radiochemical yield (RCY) > 95) in a short time over a broad range of pH values from ca. 2.4 to 9.0. Challenging experiments in the presence of excess ethylenediaminetetraacetic acid (EDTA) and in human serum revealed good stability of the [^90^Y]Y**L**^**6**^ complex. All of these experiments combined suggest the potential application of H_3_**L**^**6**^ derivatives as Y-based radiopharmaceuticals.

## Abstract 2

A new versatile asymmetric ligand based on an 18-membered macrocycle possessing three acetate pendant arms and a benzyl moiety provides fast complexation with both ^nat^Y(III) and ^90^Y(III), as well as slow dissociation kinetics. A detailed structural study in the solid state and in solution evidences nona coordination of the metal ion by the ligand, offering good protection from solvent water molecules. These favorable properties make this ligand an attractive candidate to develop yttrium-based radiopharmaceuticals.

## Introduction

Coordination chemistry plays a major role in biomedicine, as it provides an effective method for carrying metals inside living organisms allowing to take advantage of the extraordinary properties that are characteristic of some of these elements.^1^ Nevertheless, the release of free metals into the body is, to say the least, undesirable in most cases.^2,3^ Consequently, ensuring the stability of these coordination compounds is essential to guarantee their safe delivery and excretion. To achieve this goal, macrocyclic ligands are often the preferred choice when designing this type of compound, since they usually give rise to higher thermodynamic stability, as well as superior kinetic inertness.^4−7^

Undoubtedly, one of the most significant biomedical applications of macrocyclic complexes can be found in the field of biomedical imaging. With respect to the selection of metals, lanthanides have always been of paramount importance for the design of imaging agents in different techniques,^8^ and, given their unique luminescence properties (especially in the case of Eu^3+^ and Tb^3+^), their suitability for the preparation of optical probes for imaging cells, tissues, and small animals must be highlighted.^9,10^ In this case, the macrocyclic ligand should be designed not only to ensure the stability of the complex but also to maximize the photoluminescence quantum yield of the emission. As Ln^3+^ centers are known for their poor extinction coefficients, the ligand assumes the task of absorbing light through a suitable chromophore attached to its structure (commonly known as antenna), with this energy being subsequently transferred to the lanthanide ion. Additionally, it is imperative that the macrocycle wraps the metal competently to avoid solvent coordination and consequent quenching of luminescence.^11−14^

Although it cannot be considered a lanthanide in its own right, yttrium is generally included in this group and, therefore, macrocycles that coordinate effectively with lanthanide ions are usually appropriate for binding with Y^3+^.^15−17^ Nonetheless, for imaging purposes, the interest of this ion resides in its nuclear properties. Thus, the positron emitter ^86^Y has been attracting attention over the last few years as a candidate for the design of radiopharmaceuticals for positron emission tomography (PET) due to its versatile half-life (14.74 h) and its well-known chelation chemistry. Moreover, the existence of the β^–^ emitter isotope [^90^Y]-yttrium allows for the design of theranostic agents, making yttrium an extremely interesting option in nuclear medicine.^18,19^ To construct a metal-based radiopharmaceutical, a bifunctional chelator is typically chosen, which is basically a chelating ligand provided with a linker capable of conjugation to a targeting vector.^20,21^ It must be noted that time is of the essence when working with decaying nuclides, so even though kinetic inertness with respect to dissociation is still fundamental, matching the kinetics of formation with the lifetime of the radioisotope is of paramount importance as well.^22,23^

Apart from its decay properties, there are some additional nuclear attributes related to yttrium that can be found useful for medical imaging applications. The only natural isotope of yttrium, ^89^Y, presents a spin quantum number of 1/2, being therefore considered an NMR-active nucleus. Unfortunately, the extremely low gyromagnetic ratio (γ = 2.0864 MHz/T) and the long longitudinal relaxation times (*T*_1_) associated with this nucleus make the acquisition of ^89^Y NMR spectra an almost unfeasible task.^24^ Nonetheless, this weakness can be turned into strength thanks to a recently discovered technique: dynamic nuclear polarization (DNP). With the application of DNP-NMR, the unusually long *T*_1_ translates into an extended polarization lifetime of nuclear spins, which produces an extraordinary increase in sensitivity. Moreover, ^89^Y NMR spectra present sharp signals and high sensitivity of the chemical shift to the environment, making ^89^Y compounds potentially attractive as magnetic resonance imaging (MRI) probes.^25−29^

**Chart 1:**
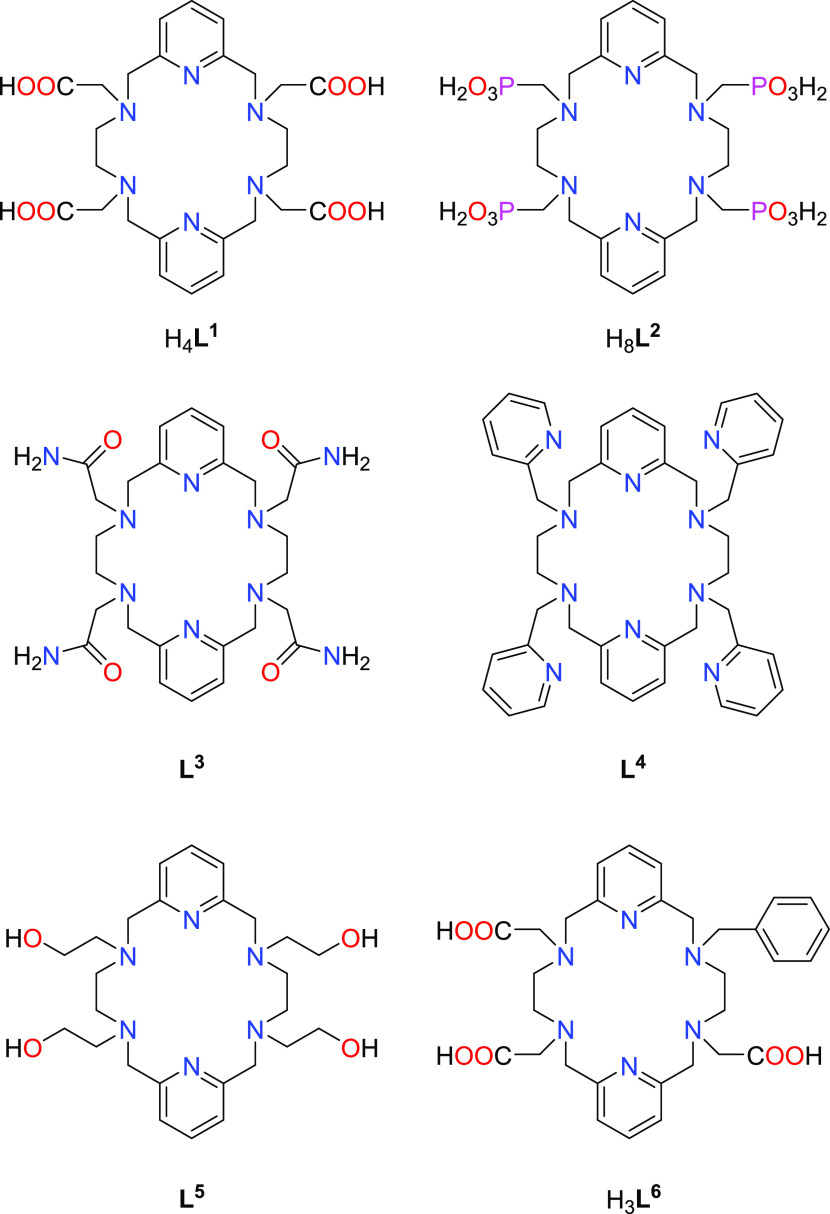
Ligands Discussed in This Work

Among the macrocyclic systems available, azamacrocycles occupy a distinguished position, given that the nitrogen donor atoms present in their structure can be easily functionalized, enabling the incorporation of pendant arms that can be used to tune and control the properties of the metal, to bind to a targeted biomolecule or simply to add additional coordination positions to increase the denticity of the ligand. Thus, the most popular macrocycles in biomedicine are those arising from the modification of the platforms tacn, cyclen, and cyclam, mainly by the inclusion of acetate pendant arms.^4^ Nevertheless, inserting pyridine moieties into the macrocyclic backbone may be worth considering since their introduction tends to increase rigidity in the ligand and to cause alterations in its basicity, leading to significant modifications in the thermodynamic and kinetic properties of its complexes.^30−33^ Accordingly, hexaazamacrocycles derived from the condensation of 2,6-diformilpyridine and ethylenediamine such as those depicted in Chart 1, have proven to successfully host lanthanide ions, due to their spacious macrocyclic cavity and their capacity to satisfy the coordination requirements of these large ions through the functionalization of their four secondary amines.^34−43^ Furthermore, it has been found that binding constants for H_4_**L**^**1**^ with large metal ions are considerably high (log *K* ∼ 22) and promising indications of kinetic inertness also exist for H_4_**L**^**1**^, **L**^**3**^, and **L**^**5**^ lanthanide complexes.^35,40,42^

Herein, we present a new nonadentate hexaazamacrocyclic ligand containing a benzyl group (H_3_**L**^**6**^) and report its coordination ability toward the Y^3+^ ion as well as the luminescence properties of its Eu^3+^ and Tb^3+^ complexes. The formation and dissociation kinetics of the Y^3+^ complex have been studied by spectrophotometric measurements. The structure of the complexes in solution was assessed using a combination of multinuclear (^1^H, ^13^C, ^89^Y) NMR spectroscopy, time-resolved emission spectroscopy, and DFT calculations. We also report the X-ray structure of the Y^3+^ and Tb^3+^ complexes. Attention should be devoted to alkylation with the benzyl moiety, which could be selectively reversed through hydrogenation. As a result, once the secondary amine is recovered, it could be functionalized a second time with a group of a different nature, such as a linker capable of bioconjugation with a relevant macromolecule, or a more efficient antenna. Another possibility for functionalization is through the para-carbon of this moiety, proving once more that this platform is quite versatile.^44^ Consequently, ligand H_3_**L**^**6**^ is expected to be a competent precursor of bifunctional chelators for Y-based radiopharmaceuticals as well as a suitable chelating agent for lanthanides for optical imaging.

## Results and Discussion

### Synthesis of the Ligand and Metal Complexes

**Scheme 1:**
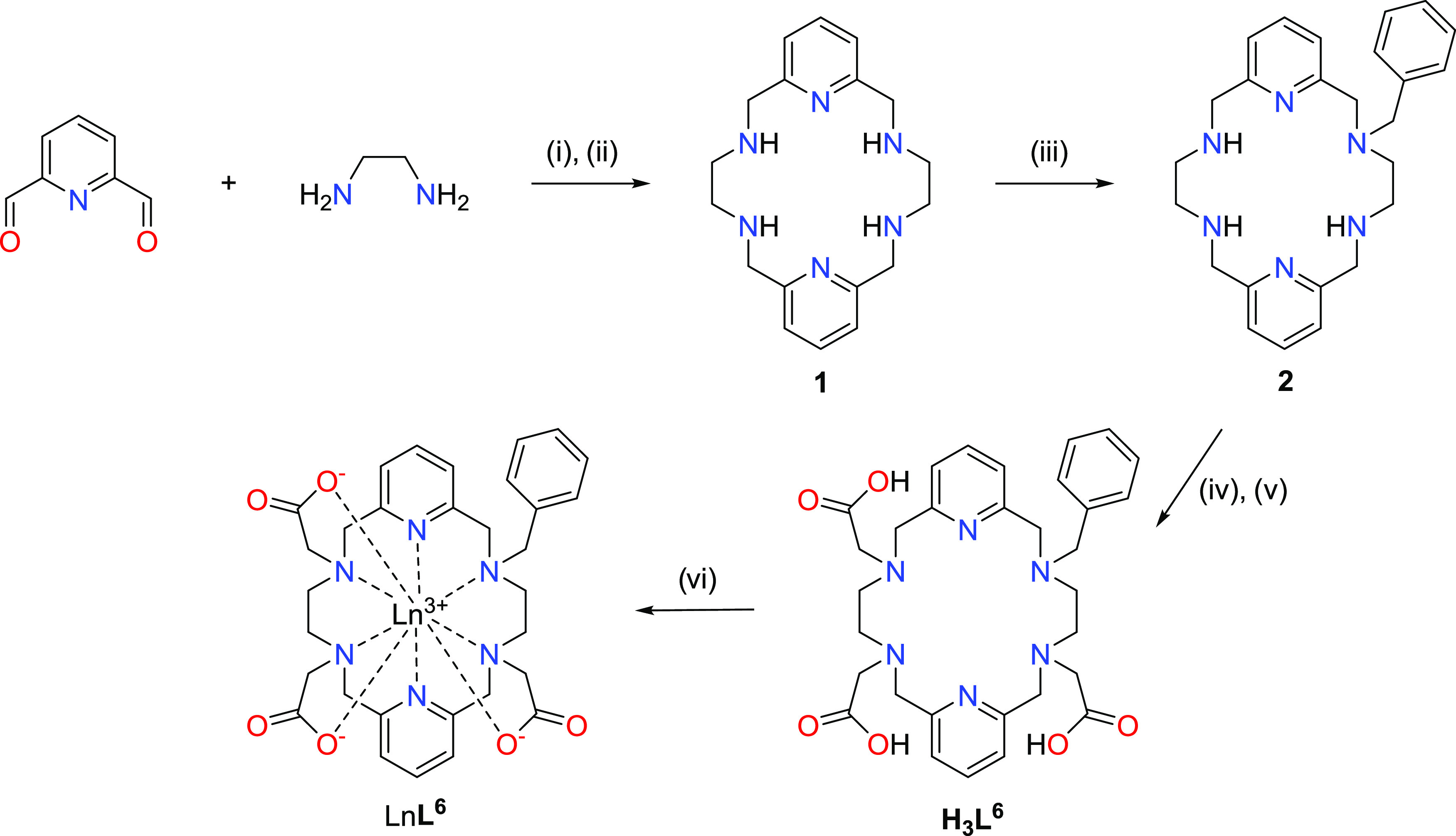
Synthetic Procedure for the Preparation of Ln**L^6^** Complexes (i) BaCl_2_, MeOH, reflux, 4 h; (ii) NaBH_4_, MeOH, 0 °C; (iii) benzyl bromide, H_2_O, pH = 5–6; (iv) BrCH_2_COO*^t^*Bu, K_2_CO_3_, CH_3_CN; (v) CF_3_COOH, CH_2_Cl_2_; (vi) Ln(OTf)_3_/YCl_3_, DIPEA, 1-butanol.

The preparation of ligand H_3_**L**^**6**^ was achieved by following the synthetic procedure described in Scheme 1. Synthesis of the parent macrocycle **1** was completed by [2 + 2] condensation of ethylenediamine and 2,6-diformylpyridine, using BaCl_2_ as a template agent, and subsequent reduction of imine moieties with sodium borohydride, as previously reported.^46^ To obtain a nonadentate ligand, asymmetric functionalization of one NH group was carried out with benzyl bromide in water under controlled pH conditions. In this way, the monosubstituted derivative, compound **2**, was obtained. This allowed the introduction of three coordinating pendants arms by *N*-alkylation with *tert*-butyl 2-bromoacetate and later hydrolysis with TFA, to finally obtain ligand H_**3**_**L**^**6**^ with a 6% overall yield starting from macrocycle **1** (three steps). This moderate result arises because of the poor yield achieved during the initial alkylation with benzyl bromide. Nevertheless, it must be taken into consideration that upon purification in this step, a large amount of unreacted parent macrocycle **1** was recovered, thereby compensating for the low efficiency of the procedure to some extent.

The reaction of H_**3**_**L**^**6**^ with Ln(OTf)_3_ (Ln = Eu or Tb) or YCl_3_ salts in 1-butanol in the presence of DIPEA as a base afforded the corresponding charge-neutral Ln**L**^**6**^ complexes in good yields (ca. 70%). The high-resolution mass spectra (ESI^+^) confirm the formation of the complexes (Figure S1, Supporting Information).

### X-ray Crystal Structure Studies

**Figure 1:**
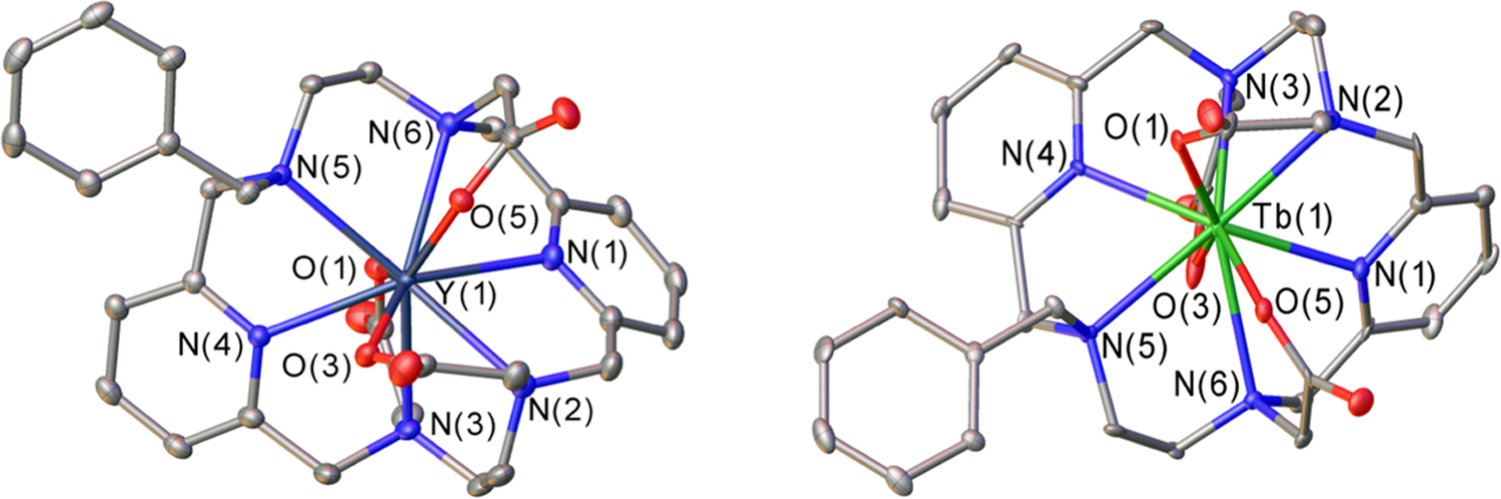
ORTEP^45^ view of the structure of the Y**L^6^** and Tb**L^6^** complexes (50% ellipsoid probability). Hydrogen atoms and water molecules are omitted for simplicity.

Slow evaporation from aqueous solutions of the Y^3+^ and Tb^3+^ complexes provided colorless block-like crystals suitable for X-ray analysis. Figure 1 displays views of the molecular structures, while bond distances of the metal coordination environments are shown in Table 1. As it can be observed, ligand H_3_**L**^**6**^ coordinates to the metal centers through the six nitrogen atoms located in the macrocyclic backbone and the three oxygen atoms from the acetate pendant arms, thus resulting in a coordination number of nine. The chelate rings formed by the coordination of the ethylenediamine moieties adopt identical conformations, which can be described as λλ or δδ. In Y**L**^**6**^, the two centrosymmetrically related enantiomers are present in the crystal lattice. Crystals of the Tb**L**^**6**^ contain both the λλ and δδ isomers in the asymmetric unit, presenting slightly different bond distances and angles. It has been shown that this type of arrangement favors the formation of a smaller macrocyclic cavity and, therefore, of shorter bonds.^38,39^ With respect to the relative disposition of the pyridyl units, it is known that ligands containing two pyridine moieties connected by an ethylenediamine bridge can present two types of conformations: the twist-wrap (tw), in which the planes that define the pyridyl entities are relatively twisted to each other, and the twist-fold (tf), where in addition to the twisting, an overall folding of the ligand over the metal is observed (Figure S2).^47^ In this case, the complex exhibits a twist-fold conformation, which is evidenced by the lack of linearity of the N(4)–Y(1)–N(1) angle (148.5°). Once again, this is not surprising, as this kind of disposition has been previously observed in similar structures displaying nine-coordinate geometry.^36^

**Table 1:** Bond Distances (Å) of the Metal Coordination Environments in Ln**L^6^** Complexes (Ln = Y or Tb)

Y(1)–O(1)	2.3019(17)	Tb(1)–O(1)	2.320(4)
Y(1)–O(5)	2.3019(15)	Tb(1)–O(5)	2.382(6)
Y(1)–O(3)	2.3097(16)	Tb(1)–O(3)	2.315(2)
Y(1)–N(1)	2.5170(19)	Tb(1)–N(1)	2.534(2)
Y(1)–N(4)	2.5191(17)	Tb(1)–N(4)	2.518(5)
Y(1)–N(5)	2.5863(16)	Tb(1)–N(5)	2.664(5)
Y(1)–N(6)	2.6249(17)	Tb(1)–N(6)	2.700(5)
Y(1)–N(3)	2.6370(18)	Tb(1)–N(3)	2.582(5)
Y(1)–N(2)	2.6574(18)	Tb(1)–N(2)	2.601(7)

**Figure 2:**
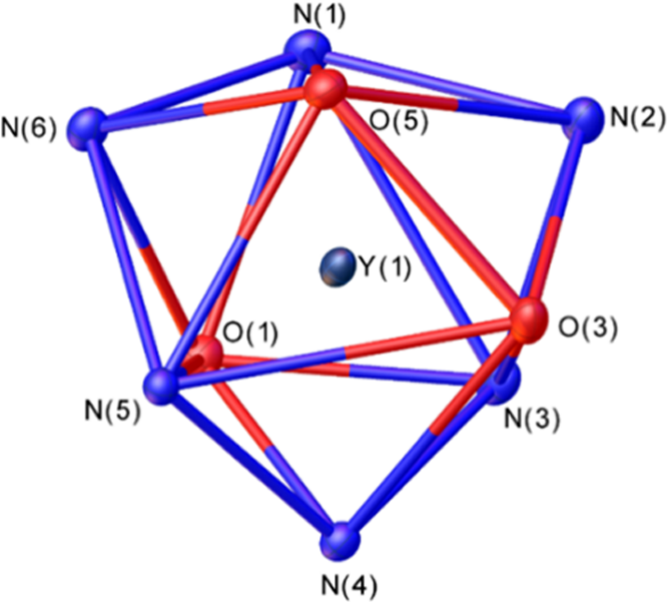
View of the tricapped trigonal prismatic coordination around the metal ion in Y**L^6^**.

The metal–N distances involving the N atoms of the pyridine units are similar to those observed for other nine-coordinated complexes of these metal ions containing pyridine units.^48−51^ The distances to the amine donor atoms of the macrocycle and the oxygen atoms of the carboxylate groups are also within the normal range observed for complexes with polyaminocarboxylate ligands.^52−55^ The coordination polyhedron around the metal ion can be best described as a tricapped trigonal prism (Figure 2). This is confirmed by the quantitative analysis carried out with the aid of the SHAPE program,^56−60^ which provides shape measures of 1.72 and 1.81 for Y**L**^**6**^ and Tb**L**^**6**^, respectively (a shape measure of 0 indicates a coordination polyhedron fully coincident with the reference polyhedron, while the maximum value of the shape measure is 100). The upper tripod of the trigonal prism is defined by the oxygen atoms of carboxylate groups O(5) and O(3) and the amine nitrogen atom N(5), while the lower tripod is delineated by N(1), N(3) and O(1). These two triangular faces are nearly parallel, intersecting at 4.0 (Y**L**^**6**^) and 4.2° (Tb**L**^**6**^). The N donor atoms (N(2), N(4), and N(6)) occupy the capping positions, defining N–(Y,Tb)–N angles in the range 117.3–122.8°, and thus are very close to the ideal values (120°).

### Photophysical Properties of the Eu and Tb Complexes

**Figure 3:**
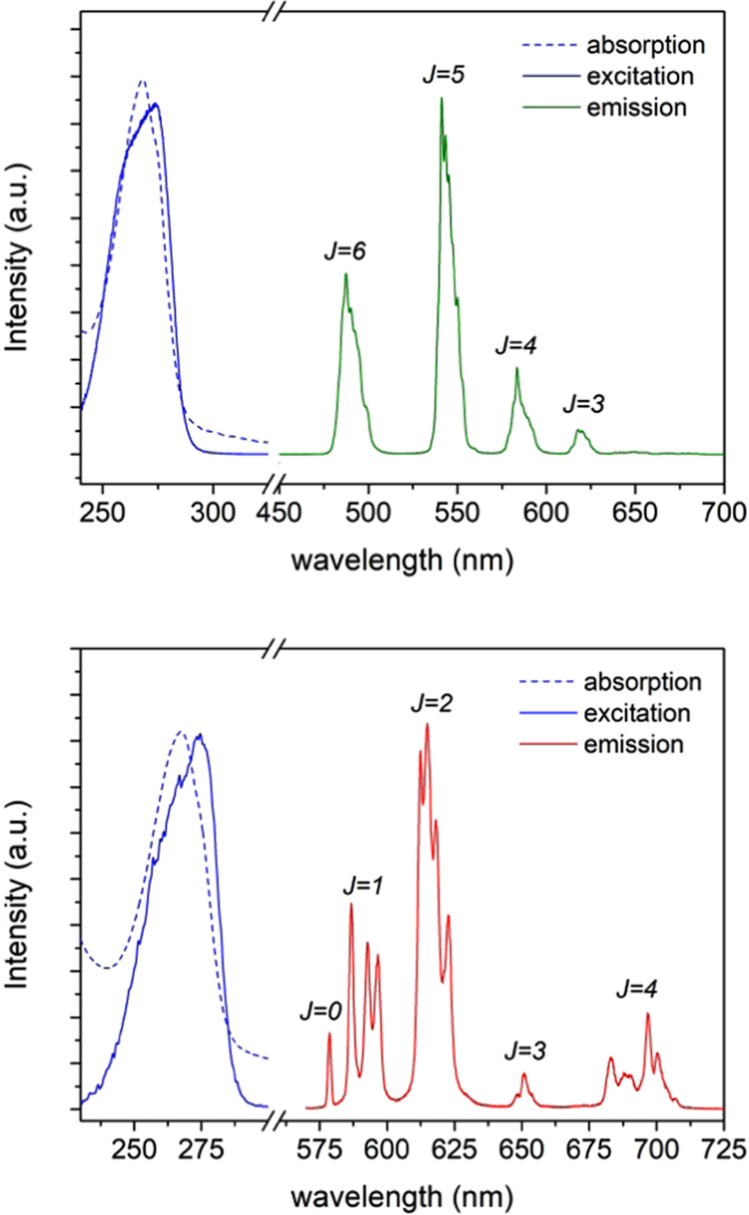
UV–vis absorption (dotted lines), excitation, and emission spectra of Tb**L^6^** (top) and Eu**L^6^** (bottom), recorded in H_2_O solution (10^–4^ M, pH ∼ 7) at room temperature.

The UV–vis absorption spectra of the Tb**L**^**6**^ and Eu**L**^**6**^ complexes in ca. 10^–4^ M aqueous solution (pH ∼ 7) are depicted in Figure 3. In both cases, the absorption spectra consist of one broad band with a maximum at 268 nm that can be assigned to the π → π* transition centered on the aromatic units of the ligand. Excitation into this absorption band led to the characteristic Ln^3+^ emission spectra displayed in Figure 3. Thus, Tb**L**^**6**^ luminescence gives rise to a set of distinctive narrow bands located between 485 and 655 nm corresponding to the metal-centered ^5^D_4_ → ^7^F_*J*_ transitions (*J* = 6–3), the most intense being placed at 542 nm (*J* = 5), as expected.^61^ On the other hand, the emission spectrum of Eu**L**^**6**^ shows an array of bands in the range of 580–710 nm, in agreement with the typical ^5^D_0_ → ^7^F_*J*_ transitions of this ion (*J* = 0–4). Particular attention must be given to the ^5^D_0_ → ^7^F_0_ transition band, whose relatively high intensity is an indication of the low symmetry of the complex. This statement is also supported by the high ^5^D_0_ → ^7^F_2_/^5^D_0_ → ^7^F_1_ intensity ratio, which is known to be strongly correlated with a low level of symmetry. In addition, it must be highlighted that the spectrum shows a single ^5^D_0_ → ^7^F_0_ transition, as can be predicted due to the nondegeneracy of the ^5^D_0_ and ^7^F_0_ levels, suggesting the existence of a single Eu^3+^ species in solution. This is in accordance with the splitting patterns observed for ^5^D_0_ → ^7^F_1_ and ^5^D_0_ → ^7^F_2_ transitions since, due to the Stark effect, they can split at most into three and five components, respectively, for a single emitting compound.^61,62^ The presence of three components for the ^5^D_0_ → ^7^F_1_ transition is also clearly indicative of a low symmetry of the crystal field created by the ligand.^62^ The ten-coordinate [Eu**L**^**3**^]^3+^ complex, which presents *D*_2_ symmetry in solution, presents two components for the ^5^D_0_ → ^7^F_1_ transition, as well as unusually intense ^5^D_0_ → ^7^F_5_ and ^5^D_0_ → ^7^F_6_ transitions that are not observed for Eu**L**^**6**^.^42^

The excitation spectra recorded for both Tb**L**^**6**^ and Eu**L**^**6**^ complexes upon metal-centered emission are very similar to the corresponding absorption spectra, which indicates that the aromatic moieties in the ligand provide an efficient energy transfer to the metal center.^64^ To determine the hydration state of Tb**L**^**6**^ and Eu**L**^**6**^ complexes, their luminescent lifetimes were measured upon emission at 617 and 542 nm, respectively, in both H_2_O and D_2_O solutions. The observed emission decays were fitted to monoexponential decay curves (Figure S3), and the resulting lifetime values were collected in Table 2. Calculation of the number of water molecules was possible through the use of Beeby^63^ and Horrocks^65^ equations, which unambiguously led to hydration numbers of zero in both cases. The lifetimes of Tb**L**^**6**^ measured in H_2_O and D_2_O solution are very similar, which leads to a small negative *q* value calculated with the expression provided by Beeby.^63^ Thus, it can be concluded that the ligand is able to satisfy the coordination requirements of these ions, preventing solvent molecules from binding to the metal center and therefore fulfilling one of the most important conditions for becoming part of a suitable fluorescent probe.

**Table 2:** Selected Photophysical Parameters for Tb**L**^6^ and Eu**L**^6^ Complexes in Aqueous Solution

	λ_max_ (ε)^a^	ϕ_H_2_O_ (%)	τ_H_2_O_ (ms)	τ_D_2_O_ (ms)	*q*^b^
Tb	268 (9900)	60	2.41	2.63	–0.1
Eu	268 (8500)	7.0	1.18	1.64	0.0

a λ_max_, nm; ε, M^–1^ cm^–1^.

b Hydration number calculated according to ref (63).

The emission quantum yields of both Tb**L**^**6**^ and Eu**L**^**6**^ have been measured in 0.1 M Tris-buffered aqueous solutions at pH = 7.4 using Eu^3+^ and Tb^3+^*tris*(dipicolinates) as standards.^66,67^ Predictably, Tb**L**^**6**^ presents a quantum yield (ϕ_H_2_O_ = 0.60) that is far superior to that observed for Eu**L**^**6**^ (ϕ_H_2_O_ = 0.07), likely because the energy of the ligand-centered triplet state presents a considerably higher energy than the emissive ^5^D_0_ level of Eu^3+^. This is indeed expected, as the excited triplet state of pyridine (32 260 cm^–1^)^68^ is much higher in energy that the ^5^D_0_ level of Eu^3+^ (∼17.240 cm^–1^), while the optimal triplet state energy for efficient energy transfer was found to be 20 000–23 000 cm^–1^.^69^ The quantum yield determined for Tb**L**^**6**^ is very high, comparable to those determined for *q* = 0 complexes containing picolinate moieties. Furthermore, the (long) lifetime of the ^5^D_4_ excited state of Tb (2.41 ms) is also close to the values reported for highly luminescent Tb^III^ complexes that lack water molecules in the inner coordination sphere.^31,70−74^

The quantum yield determined for Eu**L**^**6**^ (7%) is comparable to that of [Eu**L**^**3**^]^3+^ and represents a ∼4-fold increase with respect to [Eu**L**^**5**^]^3+^.^42,43^ This can be attributed to the quenching effect of the hydroxyl groups of the ligand coordinated to the metal ion in the latter. The [Eu**L**^**4**^]^3+^ complex displays a considerably lower quantum yield (0.1%) associated with the quenching effect of an excited charge transfer state.^43^

To gain further understanding of the energy transfer process in Eu**L**^**6**^, the metal-centered emission quantum yield was calculated following the procedure developed by Werts et al.^75^ Unfortunately, this method can only be applied to Eu^3+^ complexes, as it is based on the strong magnetic dipole nature of the ^5^D_0_ → ^7^F_1_ transition found in these compounds. Therefore, the intensity of this band can be considered independent of the chemical environment of the metal center and eq 1 can be applied for the calculation of the radiative lifetime τ_R_, where *A*_MD,0_ = 14.65 s^–1^ is the spontaneous emission probability of the ^5^D_0_ → ^7^F_1_ transition, *n* is the refractive index of the medium (1.333 for water at 589.3 nm), and *I*_tot_/*I*_MD_ is the ratio of the integrated corrected emission spectra to the area of the ^5^D_0_ → ^7^F_1_ transition.^66,73,75^
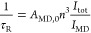
1

The value of 6.55 ms found for τ_R_ is similar to those reported in the literature for nine-coordinated Eu^III^ complexes.^76−80^ The quantum yield of the luminescence step (ϕ_Eu_) can be subsequently obtained using eq 2 since the lifetime of the Eu complex in water (τ_H_2_O_) is known (Table 2).
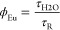
2

This analysis gives ϕ_Eu_ = 0.18, which yields a sensitization efficiency (η_sens_) of 0.39 using eq 3. This suggests that the Eu**L**^**6**^ complex presents a modest efficiency of the energy transfer taking place from the excited states of the ligand.^72,73^ Nevertheless, this analysis should be taken with caution, as *A*_MD,0_ values that depart significantly from that proposed by Werts were recently determined.^81^
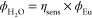
3

### Solution Structure

The diamagnetic character of Y**L**^**6**^ allowed for a more thorough analysis of its solution structure using NMR spectroscopy (^1^H, ^13^C, and ^89^Y). A rather complex ^1^H NMR spectrum was found for Y**L**^**6**^ due to the low symmetry of the molecule (*C*_1_). Nonetheless, a comparison with the spectra of the free ligand (Figure S4) corroborates the formation of the complex, not only by the chemical shifts that can be observed but also by the extensive increment in the number of signals caused by the increase in the rigidity of the molecule upon coordination. The ^13^C{^1^H} NMR spectrum (Figure S5) exhibits the 29 signals expected for a single species in solution. Interestingly, two of the signals arising from carbonyl groups appear as doublets because of coupling with ^89^Y (^2^*J*_C-Y_ ∼ 2 Hz), evidencing the coordination of the acetate groups to the metal center.

**Figure 4:**
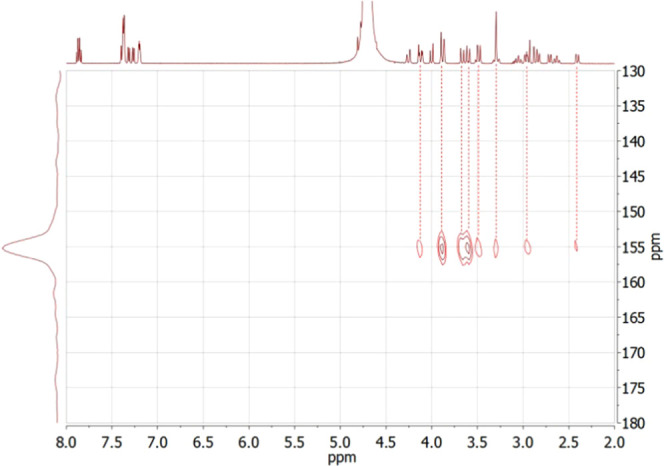
^1^H,^89^Y HMQC NMR spectrum of YL6 recorded in D_2_O solution (pH ∼ 7.0, 25 °C).

The ^89^Y NMR shift of the Y**L**^**6**^ complex was measured using ^1^H,^89^Y HMQC experiments, which provide easy access to ^89^Y NMR shifts, avoiding the long acquisition times required to obtain conventional ^89^Y NMR spectra.^24^ The ^1^H,^89^Y HMQC spectrum showed cross-peaks relating the ^89^Y nuclei with several proton nuclei of the ligand, providing an ^89^Y NMR chemical shift of 154.7 ppm (Figure 4). The ^89^Y shifts were found to be very sensitive to the number and nature of the donor atoms coordinated to the metal ion, but rather insensitive to the coordination geometry. Indeed, a relationship between the observed ^89^Y NMR shifts and nature of the donor atoms of the ligand has been established from the analysis of chemical shift data of a wide range of complexes with polyaminopolycarboxylate ligands^82^
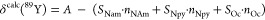
4where *A* is an empirical constant that was determined to be 863 ppm; *S*_Nam_, *S*_Npy_, and *S*_Oc_ represent the shielding contribution of amine nitrogen atoms, pyridyl nitrogen atoms, and carboxylate oxygen atoms, respectively; and *n*_Nam_, *n*_Npy_, and *n*_Oc_ are the number of donor atoms of each type. Using *S*_Nam_ = 68.1, *S*_Npy_ = 85.7, and *S*_Oc_ = 94.0, with *n*_Nam_ = 4, *n*_Npy_ = 2, and *n*_Oc_ = 3, we obtained a calculated ^89^Y shift of δ^calc^ = 137 ppm, which is in good agreement with the experimental shift. These results unambiguously confirm the coordination of the ligand to the metal ion through its N_6_O_3_ donor set and exclude the presence of coordinated water molecules (a coordinated water molecule contributes with ca. 107 ppm to the shielding of the ^89^Y resonance).^82^

DFT calculations were performed for the Ln**L**^**6**^ systems (Ln = Eu, Tb, and Y) with the purpose of understanding the geometries exhibited by these compounds. As previously stated, this type of systems can present a twist-wrap (tw) or a twist-fold (tf) conformation depending on the relative disposition of the pyridyl units. Adopting one or another can induce important changes in the properties of these compounds, and therefore a comparative study between both geometries was conducted. The calculated geometries obtained for Y**L**^**6**^ are shown in Figure S2, while bond lengths of the metal coordination spheres found for all of the systems are listed in Table S1 (Supporting Information). An excellent agreement was found for the calculated bond distances with respect to the values obtained by means of X-ray diffraction for the twist-fold conformation of the Y**L**^**6**^ complex. The calculated free energies favor the twist-fold conformation for the three complexes, with Δ*G*^o,calc^*=* Δ*G*^o^(tw) – Δ*G*^o^(tf) values of 1.8, 2.9, and 4.1 kcal mol^–1^ for Eu**L**^**6**^, Tb**L**^**6**^, and Y**L**^**6**^, respectively. Thus, the twist-fold conformation is increasingly stabilized as the ion size decreases. A quick analysis of the bond lengths reveals that the twist-fold arrangement allows for shorter bonds and this reduction with respect to the twist-wrap disposition becomes more pronounced with smaller metal centers. This is in line with the behavior mentioned above for related compounds,^38,39^ which evinces that the ligand changes its conformation as the radius of the metal ion decreases so the macrocyclic cavity is reduced, and therefore shorter bonds are favored. Relativistic DFT calculations using the DKH2 Hamiltonian (see computational details below) and the methodology described previously provided calculated ^89^Y NMR shifts of 154.6 and 120.9 ppm for the twist-fold and twist-wrap forms, respectively. The first value is in excellent agreement with the experimental value of 154.7 ppm, which confirms that the Y**L**^**6**^ complex adopts a twist-fold structure in solution.

### Dissociation and Formation Kinetics

A high stability of the complex is usually the most crucial requirement for *in vivo* applications of coordination compounds, as both the free ligand and the metal ion are often toxic. Nonetheless, even though thermodynamic parameters are important aspects to evaluate their stability, nowadays it is widely recognized that a slow dissociation of the complex is more important than a high stability constant.^23^ Acid decomplexation experiments have become a popular method to preliminarily assess the kinetic inertness of coordination compounds as well as to provide a means of comparison between different ligands. Since most complexes dissociate easily under strongly acidic conditions, the acid-catalyzed process is the main dissociation pathway found for the macrocyclic complexes usually employed for this type of applications.^51 ,72 ,83^ Accordingly, the acid-catalyzed dissociation rate of Y**L**^**6**^ was studied at 25 °C in 0.1 to 2.4 M HCl solutions. The absorption spectra of the ligand and its Y^3+^ complex are noticeably different, as a bathochromic shift can be detected upon coordination (Figure S6). Hence, the dissociation process has been studied following the variations in absorbance at 268 nm.

**Figure 5:**
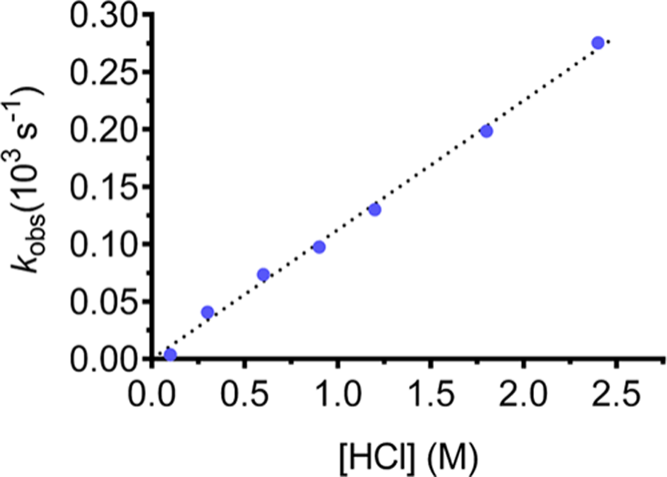
Dissociation rates (*k*_obs_) determined for Y**L^6^** as a function of HCl concentration (25 °C).

Figure 5 shows the plot of the observed dissociation rates (*k*_obs_) vs HCl concentration, which indicates the existence of a linear correlation between these two parameters. Therefore, the experimental values obtained could be fitted to eq 5
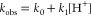
5where *k*_0_ is a constant that describes the spontaneous dissociation, while *k*_1_ characterizes the specific acid-catalysis dissociation. These results suggest that the dissociation occurs through the formation of a monoprotonated species, probably by protonation of one of the acetate pendant arms followed by proton transfer to one of the N atoms of the macrocyclic ring, subsequently displacing the metal ion from the macrocyclic cavity.^83^ The fitting procedure of the data yields a negligible (within statistical error) value for *k*_0_, which is a sign of the minor importance of spontaneous dissociation in the process, as expected under strongly acidic conditions, where protonation is favored. Consequently, the data were analyzed setting *k*_0_ to zero, obtaining a value for *k*_1_ = (1.13 ± 0.02) × 10^–4^ M^–1^ s^–1^.

The values of the rate constants listed in Table 3 indicate that Y**L**^**6**^ presents a higher kinetic inertness than YPCTA^83^ and YDO3A.^86^ The rate constant characterizing the proton-assisted dissociation pathway *k*_1_ is 1 order of magnitude lower for Y**L**^**6**^ than for YPCTA, while YDO3A is even more labile under acidic conditions. The half-lives of these complexes calculated from the rate constants confirm the higher inertness of Y**L**^**6**^ compared with YPCTA and YDO3A, indicating that Y**L**^**6**^ presents a remarkable kinetic inertness with respect to complex dissociation. The GdDOTA complex is however more inert than Y**L**^**6**^.

**Table 3:** Dissociation Rates and Half-Lives (*t*_1/2_) of Y**L^6^** and Related Complexes

	*k*_0_ (s^–1^)	*k*_1_ (M^–1^ s^–1^)	*t*_1/2_ (s)^e^
(**L**^**6**^)^3–^	0	1.13(2) × 10^–4^	6.1 × 10^3^
**PCTA**^**3**–^a	0	1.07 × 10^–3^	1.2 × 10^3^
**DOTA**^**4**–^b^,^^c^	5 × 10^–10^	2 × 10^–6^	3.5 × 10^5^
**DO3A**^**3**–^d	0	5.2 × 10^–2^	13

a Ref (83). Second-order dependence on proton-ion concentration with third-order rate constant *k*_2_ = 6.32 × 10^–4^ M^–2^ s^–1^ was observed.

b Data for the Gd complex from ref (84).

c Kinetic data for the [^90^Y]YDOTA^–^ complex at 310 K were reported in ref (85).

d Data from ref (86).

e Calculated at [H^+^] = 1 M as *t*_1/2_ = ln 2/*k*_obs_.

The rates of complexation of Y^3+^ by the **L**^**6**^ ligand were assessed in aqueous solutions buffered at pH values in the range 4.7–5.4. Pseudo-first-order conditions were ensured using an excess of the metal ion (10–40 equiv). The reaction was followed by monitoring the changes in the absorption spectrum of the ligand caused by metal complexation. The reaction was found to be very fast under these conditions, as it was nearly complete (∼90%) within only one minute (Figure S7). However, given the faint spectral changes caused by complexation, we also performed kinetic experiments using Tb^3+^ and luminescent measurements. These results confirmed a very fast complexation process, which is complete within less than one minute. Thus, these results indicate that the complexes of **L**^**6**^ are formed very quickly, in contrast to the corresponding DOTA derivatives and non-macrocyclic rigidified DTPA derivatives, as it has been shown that labeling of these ligands with ^86/90^Y-nuclides required either rather harsh conditions (heating at 75–90 °C) or extended reaction times.^23^

### Radiolabeling Experiments

**Figure 6:**
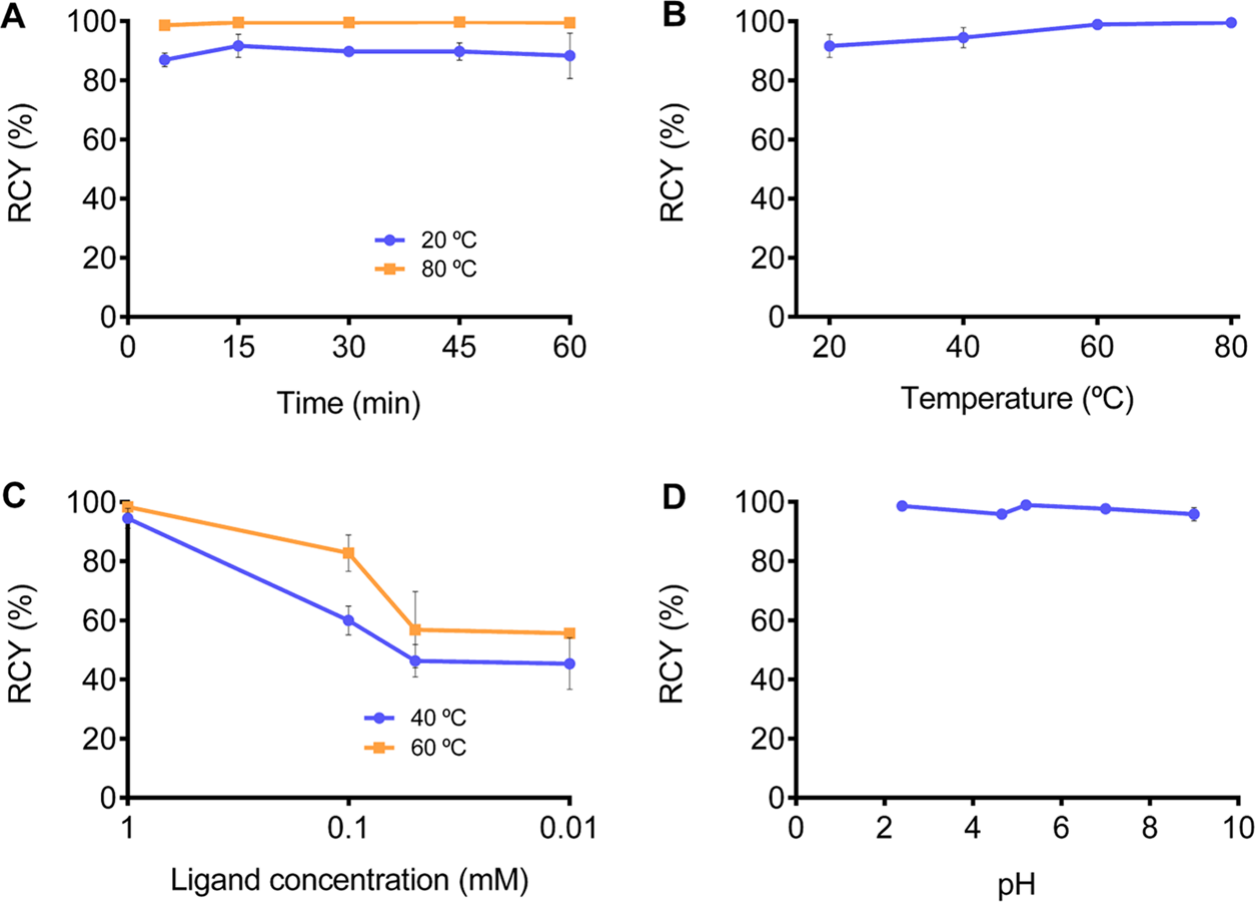
(A) ^90^Y-radiolabeling kinetics (C_L_ = 1 mM, pH 5.2, 20, and 80 °C), (B) variable temperature (C_L_ = 1 mM, pH 5.2, *t* = 15 min), (C) variation of the ligand concentration (pH 5.2, *t* = 15 min, 40 and 60 °C), and (D) pH variation, using 1 M acetate buffers (C_L_ = 1 mM, *t* = 15 min, 60 °C).

All of the results above prompted the assessment of the suitability of H_3_**L**^**6**^ for the preparation of ^90^Y-based radiopharmaceuticals. The influence of reaction conditions for radiolabeling of H_3_**L**^**6**^ with yttrium-90 was ascertained by varying the reaction time, temperature, ligand concentration, and pH (Figure 6). The ligand (1.26–126 μg; 0.002–0.2 μmol) was dissolved in ethanol and then reacted with yttrium-90 diluted in various acetate buffers. After 5 min, the radiochemical yield reached 86.9 ± 2.26% at RT and 98.6 ± 0.14% by heating to 80 °C (Figure 6A). Heating at least to 40 °C seems to be necessary to obtain a sufficient RCY but heating over 60 °C does not improve this value (Figure 6B). With a ligand concentration below 1 mM, heating to 40 °C appears to be insufficient, while heating to 60 °C leads to a better RCY (Figure 6C). [^90^Y]Y**L**^**6**^ can be synthesized over a broad range of pH (Figure 6D). Radiolabeling results confirm those obtained with ^89^Y, since [^90^Y]Y**L**^**6**^ is almost quantitatively formed in 5 min at pH = 5.2, at 60 °C (RCY = 95.5 ± 0.57%). HPLC analyses indicate [^90^Y]Y**L**^**6**^ is the sole product formed (Figure S8). Fast reaction kinetics is an advantage when preparing radiopharmaceuticals, especially if working with short-lived isotopes. The performance of H_3_**L**^**6**^ in terms of radiolabeling efficiency with the [^90^Y]Y^3+^ ion is similar to those reported for PCTA analogues containing picolinate units replacing carboxylate pendant arms.^31^ In contrast, the formation of [^90^Y]YDOTA requires heating to 60 °C at pH 7.5.^87^

### [^90^Y]YL^6^ Stability

**Figure 7:**
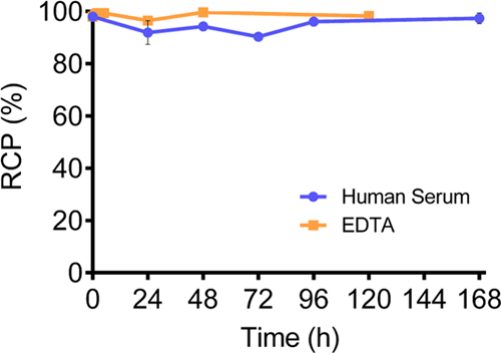
Stability of [^90^Y]Y**L**^**6**^ (10–20 μM) in an excess of EDTA (100 equiv) and in human serum.

The stability of [^90^Y]Y**L**^**6**^ in a competitive medium (ethylenediaminetetraacetic acid (EDTA) 100 mM) and in human serum was investigated. For these studies, [^90^Y]Y**L**^**6**^ was prepared using optimized conditions. A solution of [^90^Y]Y**L**^**6**^ was either diluted with an aqueous solution (v/v: 1/1) containing a large excess of the competitive EDTA ligand (100 equiv) or in 1 mL of human serum. The mixtures were incubated at 37 °C. Aliquots were taken at different time points (0, 1, 2, 5, 24, 48, and 120 h for the EDTA challenge; 0, 24, 48, 72, 96, and 168 h for serum stability) and analyzed by thin-layer chromatography (TLC). The evolution of the RCP (%) over time is represented in Figure 7.

Radiochemical purity (RCP) of [^90^Y]Y**L**^**6**^ recorded over time indicates that the complexes remain remarkably stable in the presence of EDTA. There is no dissociation or transchelation for up to 5 days, which confirms the high kinetic inertness of the Y**L**^**6**^ complex (Figure S9). Similarly, [^90^Y]Y**L**^**6**^ remains stable for over a week in the presence of human serum.

## Conclusions

We have shown that ligand H_3_**L**^**6**^ can be regarded as a suitable platform for the coordination of large metal ions such as Y^3+^, Tb^3+^, and Eu^3+^. According to the kinetic studies, the studied complexes show good inertness with respect to dissociation, making it possible to consider these compounds for biomedical applications. The remarkable kinetic inertness of these complexes is particularly striking considering the large size of the 18-membered macrocyclic unit of the ligand. Indeed, to the best of our knowledge only 12-membered macrocycles such as DOTA and PCTA, as well as certain cryptands, were found to form kinetically inert complexes with the Ln^3+^ ions (and Y^3+^). Thus, the results reported in this work pave the way for a new generation of macrocyclic ligands for the stable complexation of these metal ions. In addition, the formation rates of the Y**L**^**6**^ complex exceed those of YDOTA and rigidified YDTPA derivatives,^23^ and therefore ligand H_3_**L**^**6**^ could be especially valuable as a precursor for the design of yttrium-based radiopharmaceuticals.

On the other hand, according to the X-ray analyses and the calculations shown in this work, the resulting complexes tend to adopt geometries that favor the reduction of the macrocyclic cavity. Consequently, the ligand effectively wraps the metal ion, hindering the entrance of solvent molecules into the coordination sphere. As a result, quenching of luminescence is minimized for the Tb**L**^**6**^ and Eu**L**^**6**^ complexes, therefore meeting the key requirements for optical imaging applications.

In summary, ligand H_3_**L**^**6**^ exhibits appropriate characteristics to be considered a useful platform for the design of different types of diagnostic and/or therapeutic probes. Furthermore, the possibility of functionalizing the ligand through the benzyl moiety or replacing it with other groups expands the range of possible specific applications for systems derived from this one, resulting in a remarkably versatile ligand.

## Experimental and Computational Section

### General Considerations

NMR spectra were obtained at 25 and 70 °C on a Bruker Avance 300, Bruker Avance 400, or Bruker Avance 500 spectrometer. Elemental analyses were performed on a Thermo Finnigan Flash EA 1112 elemental analyzer. IR spectra were recorded using a Thermo Scientific FT-IR Nicolet iS10 spectrophotometer equipped with a Thermo Scientific Smart iTR attenuated total reflectance (ATR) accessory. Mass spectra were obtained either using an LC-Q-q-TOF Applied Biosystems QSTAR Elite spectrometer or an LTQ-Orbitrap Discovery mass spectrometer coupled to a Thermo Accela HPLC in ESI positive mode. Medium performance liquid chromatography (MPLC) was performed in a Puriflash XS 420 InterChim Chromatographer equipped with a UV-DAD detector and a 20 g BGB Aquarius C18AQ reversed-phase column (100 Å, spherical, 15 μm). Aqueous solutions of the final compounds were lyophilized in a Biobase BK-FD10 Series vacuum freeze dryer.

### Absorption and Emission Spectra

UV–vis spectra were recorded on a Jasco V-650 spectrophotometer using 1 cm cells. Emission and excitation spectra were measured on a Horiba FluoroMax Plus-P spectrofluorometer equipped with a 150 W ozone-free xenon arc lamp and an R928P photon counting emission detector, as well as a photodiode reference detector for monitoring lamp output. Luminescence decays were measured on the same instrument working in the phosphorescence mode using a xenon flash lamp. Hydration numbers (*q*) were calculated using eq 6, where τ_H_2_O_ and τ_D_2_O_ represent the luminescence decay lifetimes in water and deuterated water, respectively. For Tb**L**^**6**^, the hydration number was calculated using *A* = 5.0 and *B* = 0.06,^63^ while for Eu**L**^**6**^, the reported hydration number corresponds to that obtained with *A* = 1.2 and *B* = 0.25.^63^ The use of *A* = 1.11 and *B* = 0.31 provides essentially the same result (*q* = −0.08).^65^
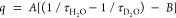
6

Luminescence quantum yields were obtained using Eu^3+^ and Tb^3+^ tris(dipicolinates) as references in solutions at 7.5 × 10^–5^ and 6.5 × 10^–5^ M, respectively (ϕ^Eu^ = 0.24 and ϕ^Tb^ = 0.22, λ_exc_ = 279 nm), while samples were measured at 1 × 10^–4^ M. Both the samples and the references were measured in 0.1 M Tris-buffered aqueous solutions at pH = 7.4.^66,67^

### Dissociation and Formation Kinetics

Acid-catalyzed dissociation kinetics of Y**L**^**6**^ were studied under pseudo-first-order conditions by the addition of concentrated HCl to an aqueous solution of the complex (2 × 10^–5^ M) at 25 °C. HCl concentration was varied in the range 0.1–2.4 M. Dissociation was followed by monitoring the decrease of the absorbance at 268 nm as a function of time using a Biochrom Libra S70 UV–vis spectrophotometer. The data were fitted to eq 7
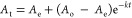
7where *A*_t_*, A*_e_, and *A*_o_ are the absorbance values measured at time *t*, at equilibrium, and at *t* = 0, respectively.

The formation of the Y**L**^**6**^ complex was assessed by the addition of an excess of metal ion (10–40 equiv) to an aqueous solution of the ligand at 2 × 10^–5^ M and following the increase in absorbance at 275 nm over time until equilibrium was reached. Similarly, the formation of Tb**L**^**6**^ was monitored by analyzing the emission intensity at 541 nm over time, with the aid of an SLM AMINCO Bowman series 2 luminescence spectrometer. The studies were performed in both cases at 25 °C with ionic strength adjusted to 0.1 M with KCl and *N*-methylpiperazine as a buffer to maintain the pH constant (4.7–5.4).

### Syntheses

All solvents and reagents used were purchased from commercial sources, had reagent-grade quality, and were used as supplied, without further purification except for macrocycle **1**, which was prepared according to the previously reported procedure.^46^

#### 3-Benzyl-3,6,10,13-tetraaza-1,8(2,6)-dipyridinacyclotetradecaphane (**2**)

Compound **1** (0.2737 g, 0.84 mmol) was suspended in H_2_O (100 mL). The pH was lowered to 5 using 6 M HCl. As the pH is lowered, compound **1** dissolves completely. Benzyl bromide (0.1434, 0.84 mmol) was slowly added to the mixture, forming a suspension. The reaction mixture was then kept stirring at room temperature for 11 days, maintaining the pH between 5 and 6. The solvent was removed in a rotary evaporator to give a brown oil, which was dissolved in a mixture of H_2_O containing 0.1% of TFA (2 mL) and purified by MPLC using Method A (Table S2, Supporting Information). Compound eluted at 41% CH_3_CN, (retention time: 9.18 column volumes, 11:53 min:s). The combined fractions containing compound **2** were then freeze-dried obtaining 74.1 mg of a hygroscopic white-brown solid. Yield: 21%. ^1^H NMR (300 MHz, D_2_O): δ 8.0 (t, 1H), 7.9 (t, 1H), 7.4 (m, 9H), 4.6 (m, 6H), 4.5 (s, 2H), 4.4 (s, 2H), 3.8 (m, 8H). ^13^C{^1^H} NMR (75 MHz, D_2_O): δ 150.40, 150.23, 150.21, 149.83, 139.75, 139.62, 131.29, 129.31, 129.04, 128.15, 124.25, 123.03, 122.94, 58.38, 56.91, 50.91, 50.79, 43.91, 43.85, 42.31. MS (ESI^+^, %BPI): *m*/*z* 417.277 (100) ([C_25_H_33_N_6_]^+^), 439.258 (26) ([C_25_H_32_N_6_Na]^+^). Calc. for [C_25_H_33_N_6_]^+^: 417.276; [C_25_H_32_N_6_Na]^+^: 439.258.

#### 2, 2′, 2″-(13-Benzyl-3,6,10,13-tetraaza-1,8(2,6)-dipyridinacyclotetradecaphane-3,6,10-triyl) Triacetic Acid (H_3_L^6^)

Compound **2** (0.065 g, 0.156 mmol) was dissolved in CH_3_CN (20 mL). K_2_CO_3_ (0.067 g, 0.484 mmol) and KI (2.59 mg, 0.0156 mmol) were added to the resulting mixture and, after stirring for 30 min, a solution of *tert*-butyl 2-bromoacetate (0.0913 g, 0.468 mmol) in acetonitrile (5 mL) was added dropwise over the course of 1 h. The mixture was stirred at room temperature for 3 days and then was concentrated to dryness. The resulting residue was dissolved in water (30 mL) and the pH of the solution was adjusted with NaOH to an approximate value of 13. This aqueous solution was extracted with chloroform (3 × 30 mL). The combined organic extracts were dried over Na_2_SO_4_ and evaporated to dryness, obtaining a brown oil. The resulting product was dissolved in a 1:1 CH_2_Cl_2_/TFA solution (3 mL) and stirred for 24 h at room temperature. The solvent was removed under a flow of nitrogen obtaining a brown residue that was washed with water (6 × 3 mL). This residue was then purified by reversed-phase MPLC method B (Table S3, Supporting Information). Compound eluted at 35% CH_3_CN, (retention time: 8.86 column volumes, 12:07 min:s). The combined fractions were freeze-dried obtaining 0.0392 g of H_3_**L**^**6**^ as a white powder. Yield: 28%. ^1^H NMR (400 MHz, D_2_O, pD = 1.0, 343 K) δ 8.8 (t, 1H), 8.4 (t, 1H), 8.2 (d, 2H), 7.9 (d, 1H), 7.8 (m, 6H), 5.2 (s, 2H), 5.0 (m, 6H), 5.0 (s, 2H), 4.4 (s, 2H), 4.3 (d, 6H), 4.1 (d, 4H), 4.1 (m, 2H). ^13^C{^1^H} NMR (126 MHz D_2_O, pD 1.0): δ 172.66, 170.82, 150.71, 150.18, 148.10, 140.03, 131.68, 130.32, 128.84, 127.53, 126.09, 124.84, 123.69, 123.16, 59.87, 58.80, 56.95, 56.61, 55.98, 55.71, 54.91, 53.26, 52.01, 50.33, 50.02. Elem. anal. found: C, 48.20; H, 4.48; N, 9.23. Calc. for C_31_H_38_N_6_O_6_·2.8TFA: C, 48.31; H, 4.52; N, 9.24. IR (ATR, cm^–1^): ν 2924, 2853 (C–H), 1725 (C=O), 1667 (C=N), 1457, 1397 (C=C), 1173, 1127 (C–O). MS (ESI^+^, %BPI): *m*/*z* 591.293 (100) ([C_31_H_39_N_6_O_6_]^+^), 629.240 (19) ([C_31_H_38_KN_6_O_6_]^+^), 613.275 (18) ([C_31_H_38_NaN_6_O_6_]^+^). Calc. For [C_31_H_39_N_6_O_6_]^+^: 591.293; [C_31_H_38_KN_6_O_6_]^+^: 629.248; ([C_31_H_38_NaN_6_O_6_]^+^): 613.275.

### General Procedure for the Preparation of LnL^6^ Complexes

A mixture of H_3_**L**^**6**^ (0.075 g, 0.067 mmol) and DIPEA (0.063 g, 0.49 mmol) in 1-butanol (6 mL) was stirred for 30 min. Ln(OTf)_3_ (Ln = Eu, Tb) or YCl_3_·6H_2_O (0.067 mmol) was added to the solution, and the mixture was heated to reflux for 8 h. The solvent was removed in a rotary evaporator, and the resultant residue was purified by column chromatography (SiO_2_, CH_3_CN/H_2_O 14:3). The product obtained was dissolved in CH_3_CN (20 mL) and passed through a filter with a 0.22 μm pore size. The filtrate was concentrated to dryness and washed with diethyl ether.

**EuL**^**6**^**:** Yield: 0.036 g, 70%. Elem. anal. found: C, 46.64; H, 4.84; N, 9.32. Calc. for C_31_H_35_EuN_6_O_6_·3H_2_O: C, 46.91; H, 5.21; N, 10.59. MS (ESI^+^, %BPI): *m*/*z* 763.17 (100) ([C_31_H_35_EuN_6_NaO_6_]^+^), 741.19 (48) ([C_31_H_36_EuN_6_O_6_]^+^). HR-MS (ESI^+^): 741.1930. Calc. for [C_31_H_36_EuN_6_O_6_]^+^: 741.1903.

**TbL**^**6**^**:** Yield: 0.040 g, 74%. Elem. anal. found: C, 45.71; H, 5.25; N, 9.70. Calc. for C_31_H_35_N_6_O_6_Tb·4H_2_O: C, 45.48; H, 5.29; N, 10.27. MS (ESI^+^, %BPI): *m*/*z* 769.18 (100) ([C_31_H_35_N_6_NaO_6_Tb]^+^), 747.20 (20) ([C_31_H_36_N_6_O_6_Tb]^+^). HR-MS (ESI^+^): 747.1965. Calc. for [C_31_H_36_N_6_O_6_Tb]^+^: 747.1944.

**YL**^**6**^**:** Yield: 0.033 g, 66%. ^1^H NMR (D_2_O, pD = 7.5, 300 MHz): δ 7.92 (m, 2H), 7.46-7.24 (m, 9H), 4.86 (m, 1H), 4.35–3.91 (m, 7H), 3.74–3.52 (m, 4H), 3.35 (m, 3H), 3.16–2.63 (m, 7H), 2.46 (d, 1H), 1.29 (d, 1H). ^13^C{^1^H} NMR (D_2_O, pD = 7.5, 125.8 MHz): δ 181.5 (d, ^2^*J* = 2.1 Hz), 180.5, 179.5 (d, ^2^*J* = 2.0 Hz), 159.7, 159.4, 156.0, 155.1, 141.4, 140.5, 131.9, 131.4, 128.6, 128.4, 122.9, 122.6, 122.2, 120.1, 65.6, 64.8, 64.3, 63.1, 62.0, 61.7, 58.8, 57.9, 57.6, 54.9, 54.4, 53.1. Elem. anal. found: C, 49.61; H, 5.13; N, 10.43. Calc. for C_31_H_35_N_6_O_6_Y·4H_2_O: C, 49.74; H, 5.79; N, 11.23. MS (ESI^+^, %BPI): *m*/*z* 699.16 (100) ([C_31_H_35_N_6_NaO_6_Y]^+^), 677.18 (14) ([C_31_H_36_N_6_O_6_Y]^+^).HR-MS (ESI^+^): 677.1756. Calc. for [C_31_H_36_N_6_O_6_Y]^+^: 677.1749.

### Crystal Structure Determinations

Crystallographic data were collected at 100 K using a Bruker D8 Venture diffractometer with a Photon 100 CMOS detector and Mo Kα radiation (λ = 0.71073 Å) generated by an Incoatec high-brilliance microfocus source equipped with Incoatec Helios multilayer optics. The software APEX3^88^ was used for collecting frames of data, indexing reflections, and the determination of lattice parameters, SAINT^89^ for integration of intensity of reflections, and SADABS^90^ for scaling and empirical absorption correction. The structure was solved by dual-space methods using the program SHELXT.^91^ All nonhydrogen atoms were refined with anisotropic thermal parameters by full-matrix least-squares calculations on F^2^ using the program SHELXL-2014.^92^ Hydrogen atoms were inserted at calculated positions and constrained with isotropic thermal parameters. The OLEX2 solvent mask routine was used to delete highly disordered water molecules from the model in both structures. CCDC 2143076 and 2143077 contain the supplementary crystallographic data for Y**L**^**6**^ and Tb**L**^**6**^ respectively. These data can be obtained free of charge from the Cambridge Crystallographic Data Centre via www.ccdc.cam.ac.uk/data_request/cif. Table S4 contains the crystallographic data and the structure refinement parameters.

### Computational Details

Full-geometry optimizations of the complexes studied in this work were performed using DFT within the hybrid meta-generalized gradient approximation with the TPSSh exchange–correlation functional^93^ and the Gaussian 09 package (Revision D.01).^94^ The ligand atoms were described using the standard 6-31G(d,p) basis set, while for the metal ions, an effective core potential (ECP) was employed to take into account the main relativistic effects and reduce the computational cost of the calculations. In the case of yttrium, the quasi-relativistic effective core potential ECP28MWB developed by Preuß and co-workers was used, along with its associated valence-basis set, which employs an (8s7p6d2f1g)/[6s5p3d2f1g]-GTO contraction scheme.^95,96^ The lanthanide ions were defined using the large-core quasi-relativistic effective core potential (LCRECP) created by Dolg and co-workers, which includes 46 + 4f^n^ core electrons in the core (*n* = 6 for Eu^3+^ and *n* = 8 for Tb^3+^) and explicitly describes the 11 outer electrons (5s, 5p, 5d, and 6s). The valence electrons were described using the associated (7s 6p 5d)/[5s 4p 3d]-GTO basis set.^97^ The calculations were carried out in aqueous solutions and solvent effects were included making use of the integral-equation formalism variant of the polarizable continuum model (IEFPCM).^98^ As a starting point, molecular systems generated with GaussView^99^ were employed. Additionally, frequency analyses were performed on the optimized geometries to guarantee that they indeed correspond to energy minima rather than saddle points.

Using these optimized geometries, the ^89^Y NMR shielding tensors were calculated with the ORCA program package (version 4.2.1)^100,101^ utilizing the GIAO^102,103^ method and the TPSSh functional.^93^ Relativistic effects were considered applying the second-order Douglas–Kroll–Hess (DKH2) method,^104,105^ with the old-DKH-TZVPP basis set used by previous versions of ORCA consisting in a recontracted form of Ahlrichs’ TZVPPAll basis set^106^ for DKH2 calculations. The RIJK approximation, which considers both Coulomb and exchange-type integrals, was used for the calculation of the self-consistent field and the NMR chemical shielding constants.^107−109^ Auxiliary basis sets were constructed automatically by ORCA with the Autoaux procedure.^110^ The TightSCF and Grid7 (for Y) options were applied to increase the convergence tolerances and integration accuracies of the calculations from the defaults. Chemical shifts were determined as δ = (σ^ref^ – σ) considering the shielding constant calculated for [Y(H_2_O)_8_]^3+^·16H_2_O as in ref (41). The calculations were carried out in aqueous solution and solvent effects were considered using the SMD solvation model.^111^

### Radiolabeling Studies

Yttrium-90 chloride ([^90^Y]YCl_3_) was provided by PerkinElmer Life Sciences (Waltham, MA) in a 0.05 M HCl solution. The activity of the ^90^Y-solution comprised between 200 μCi and 1.2 mCi (7.5–45.5 MBq). Other chemicals (solvents, buffer solutions) were bought from Sigma-Aldrich (Saint-Louis, MO) and used as received. Experiments were performed in borosilicated sealed glass flasks. Sealed flasks were heated on a Bioblock heating block (Thermo Fisher, Waltham, MA). Activities were measured with a CRC-127R (Capintec, Inc., Ramsey, NJ) dose calibrator. Radiochemical yields (RCY) were determined by thin-layer chromatography (TLC) on Whatman 1 paper (GE Healthcare, Maidstone, U.K.) eluted in MeOH with 0.1% NEt_3_ and measured with a Cyclone Storage Phosphorimager (PerkinElmer, Waltham, MA), using the Optiquant software. HPLC analyses were performed on an HPLC Dionex Ultimate 3000 (Sunnyvale, CA) equipped with a diode array detector and a radiochromatographic *f*Lumo (Berthold Technologies GmbH, Bad Wildbad, Germany) detector piloted by the Chromeleon software. The chromatographic analytic system employs an Accucore C_18_ 100 × 3 mm^2^, 2.6 μm column with A = H_2_O; B = acetonitrile as eluents; 0–3 min: 100% A, 3–20 min: 0–90% B, 20–25 min: 90% B, 25–26 min: 90–0% B, 26–30 min: 100% A, at a flow rate of 0.4 mL/min.

### ^90^Y-Radiolabeling

Yttrium-90 is a pure high-energy β-emitting nuclide. Experiments were done in a controlled area adapted for the manipulations of such elements, by trained and suitably equipped and monitored operators (finger and chest dosimeters, direct reading personal device). Operations were done inside a high-energy hotcell, using dedicated high-energy tungsten shielding for vials, syringes, and telescopic pliers. Several parameters such as concentration of ligands, volume and pH of the reaction mixture, incubation time, and temperature were varied extensively to obtain an optimized protocol. An [^90^Y]YCl_3_ solution (0.2 mL) in 1 M glacial acetic acid solution (pH = 2.4) or in 3 M acetate buffer (pH = 4.65–9) was added to 0.2 mL of H_3_**L**^**6**^ ligand solution (*c* = 10 μM–1 mM) in ethanol. The resulting solution was heated at 20–100 °C for 5–60 min.

### Stability of the [^90^Y]YL^6^ Radiochelate

For the challenging experiments, aliquots (0.2 mL) of [^90^Y]Y**L**^**6**^ solution prepared under an optimized procedure were mixed with 0.2 mL of a 100 mM EDTA solution. The mixture was incubated at 37 °C under slight stirring and analyzed on TLC after 0, 1, 2, 5, 24, 48, and 120 h. Each sample was analyzed in triplicate.

For the stability study in serum, aliquots (0.2 mL) of [^90^Y]Y**L**^**6**^ solution prepared under optimized procedure were mixed with 1 mL of a human serum. The mixture was incubated at 37 °C under slight stirring. A 100 μL aliquot was taken and, after denaturing serum protein with an equal amount of absolute ethanol and centrifugation (3500*g*, 4 °C, 15 min), the supernatant was analyzed on TLC after 24, 48, 72, 96, and 168 h. Each sample was analyzed in triplicate.

## Supporting Information Available

Luminescence lifetime decay curves; ^1^H, ^13^C, and ^1^H–^1^H COSY NMR spectra; high-resolution MS; kinetic experiments; absorption spectra; bond distances; and optimized geometries obtained with DFT (PDF)

## Accession Codes

CCDC 2143076–2143077 contain the supplementary crystallographic data for this paper. These data can be obtained free of charge via www.ccdc.cam.ac.uk/data_request/cif, or by emailing data_request@ccdc.cam.ac.uk, or by contacting The Cambridge Crystallographic Data Centre, 12 Union Road, Cambridge CB2 1EZ, UK; fax: +44 1223 336033.
