# Supplementary material for: Versatile Macrocyclic Platform for the Complexation of [natY/90Y]Yttrium and Lanthanide Ions
Source: Inorg Chem. 2022 Apr 14;61(16):6209–22. doi: 10.1021/acs.inorgchem.2c00378 (PMC9044452; doi:10.1021/acs.inorgchem.2c00378)
Supplement: Supplementary file 1 — ic2c00378_si_001.pdf [file ic2c00378_si_001.pdf]

Supporting Information for:

A Versatile Macrocyclic Platform for Complexation of [^{nat}Y/^{90}Y]Yttrium and Lanthanide Ions

Charlene Harriswangler,† Laura Caneda Martínez,† Olivier Rousseaux,‡ David Esteban-Gómez,† Olivier Fougère,‡ Rosa Pujales-Paradela,† Laura Valencia,§ M. Isabel Fernández,† Nicolas Lepareur,|| and Carlos Platas-Iglesias,*, †

† Universidade da Coruña, Centro de Investigacións Científicas Avanzadas (CICA) and Departamento de Química, Facultade de Ciencias, 15071, A Coruña, Galicia, Spain

‡ Groupe Guerbet, Centre de Recherche d'Aulnay-sous-Bois, BP 57400, 95943 Roissy CdG Cedex, France

§ Departamento de Química Inorgánica, Facultad de Ciencias, Universidade de Vigo, As Lagoas, Marcosende, 36310 Ponte-vedra, Spain

|| Univ Rennes, Centre Eugène Marquis, Inrae, Inserm, Institut NUMECAN (Nutrition, Métabolismes et Cancer) – UMR_A 1341, UMR_S 1241, F-35000 Rennes, France

Summary

Figure S1. Calculated (red) and observed (blue) mass spectral isotopic distribution for the fragments $[EuL^6+H]^+$ (top left), $[TbL^6+H]^+$ (top right) and $[YL^6+Na]^+$ (bottom). 1

Figure S2. Calculated structures for the *twist-wrap* (left) and *twist-fold* (right) conformations of complex YL^6. 1

Figure S3. Variation of the emission intensity observed over time in H_2O (blue) and D_2O (orange) solutions for 10^{-5} M TbL^6 (left) and 10^{-5} M EuL^6 (right) measured at 542 and 617 nm, respectively. 2

Figure S4. Comparison between the ^1H-NMR spectra of ligand H_3L^6 (at 298 and 343 K) and its Y^{3+} complex. 2

Figure S5. $^{13}C\{^1H\}$-NMR spectrum of YL^6 and detail of the splitting of one of the carbonyl signals due to its coupling with the metal center. 3

Table S1. Bond distances (Å) of the coordination environment of the metal (M) for the *twist-wrap* (tw) and *twist-fold* (tf) conformations of the complexes. 3

Figure S6. Comparison between UV/Vis absorption spectra of the complex YL^6 (orange, dashed) and the ligand H_3L^6 (blue) at pH 7.0 and concentration 2 x 10^{-5} M. 4

Figure S7. Time course of the complexation of ligand L^6 (2 x 10^{-5} M) with Y^{3+} (orange) and Tb^{3+} (green) measured after the addition of 10 equivalents of metal at pH 5.4 in N-methylpiperazine buffered solution 4

Figure S8. HPLC chromatogram of $[^{90}Y]YL^6$. 5

Table S2. MPLC purification method A. 6

Table S3. MPLC purification method B. 6

Table S4. Crystal Data and Structure Refinement Details. 7

Figure S10. ^1H-NMR spectrum of compound 2 (300 MHz, D_2O, 298 K) 8

Figure S11. $^{13}C\{^1H\}$-NMR spectrum of compound 2 (75 MHz, D_2O, 298 K) 8

Figure S12. Experimental high resolution mass spectrum (ESI^+) of compound 2 9

Figure S13. ^1H-NMR spectrum of H_3L^6 (500 MHz, D_2O, 298 K, pH ~ 1) 9

Figure S14. High temperature ^1H-NMR spectrum of H_3L^6 (400 MHz, D_2O, 343 K, pH ~ 1). 10

Figure S15. $^{13}C\{^1H\}$-NMR spectrum of H_3L^6 (126 MHz, D_2O, 298 K, pH ~1) 10

Figure S16. Experimental high resolution mass spectrum (ESI^+) of compound L^6 11

Figure S17. ^{1}H-^{1}H COSY NMR spectrum of YL^6 recorded in D_2O solution (10 mM)..11

YL^6 *twist-wrap* TPSSh/ECP28MWB/6-31G(d,p), aqueous solution (IEFPCM), 0 imaginary frequencies 12

YL^6 *twist-fold* TPSSh/ECP28MWB/6-31G(d,p), aqueous solution (IEFPCM), 0 imaginary frequencies 14

EuL^6 *twist-wrap* TPSSh/LCRECP/6-31G(d,p), aqueous solution (IEFPCM), 0 imaginary frequencies 16

EuL^6 *twist-fold* TPSSh/LCRECP/6-31G(d,p), aqueous solution (IEFPCM), 0 imaginary frequencies..18

TbL^6 *twist-wrap* TPSSh/LCRECP/6-31G(d,p), aqueous solution (IEFPCM), 0 imaginary frequencies 20

TbL^6 *twist-fold* TPSSh/LCRECP/6-31G(d,p), aqueous solution (IEFPCM), 0 imaginary frequencies..22

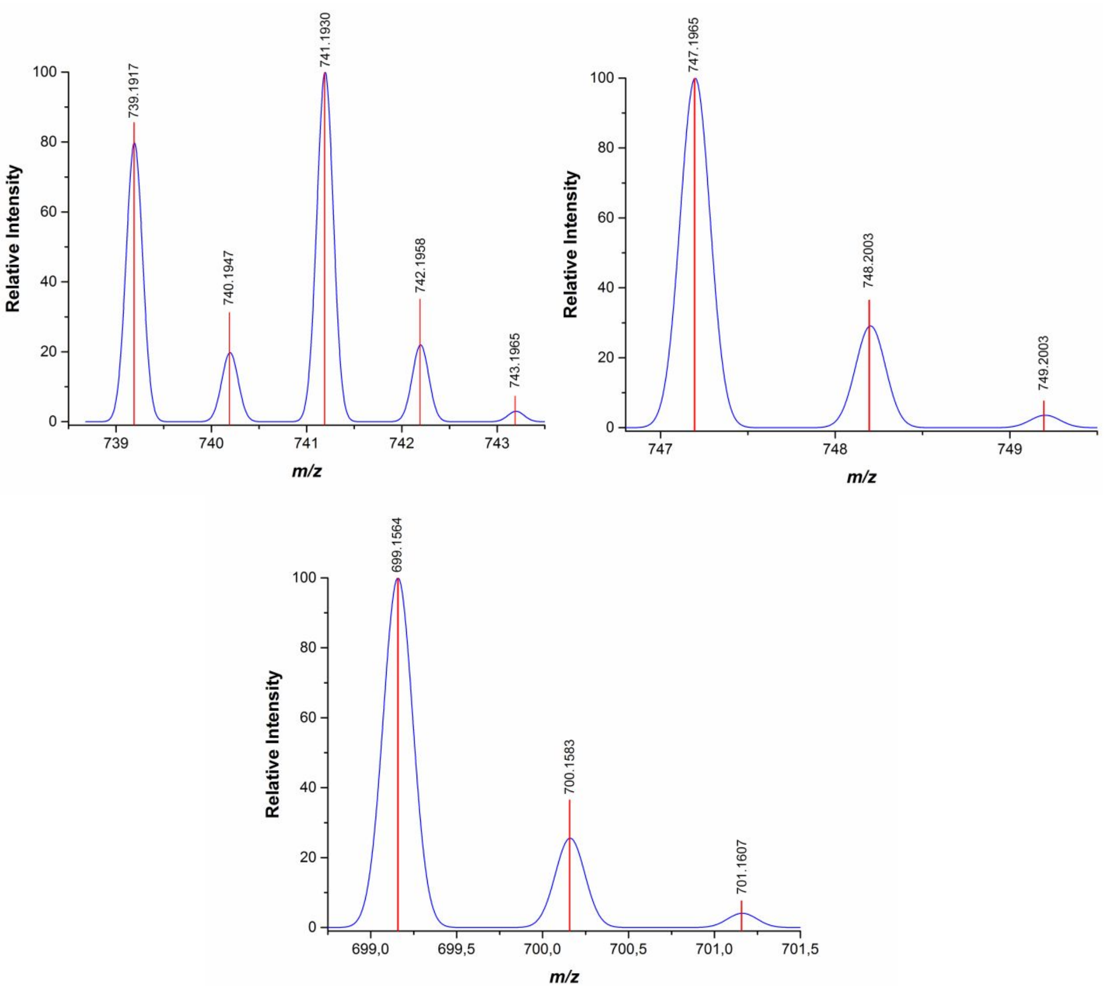

Figure S1. Calculated (red) and observed (blue) mass spectral isotopic distribution for the fragments [Eu**L**^{6}+H]$^{+}$ (top left), [Tb**L**^{6}+H]$^{+}$ (top right) and [Y**L**^{6}+Na]$^{+}$ (bottom).

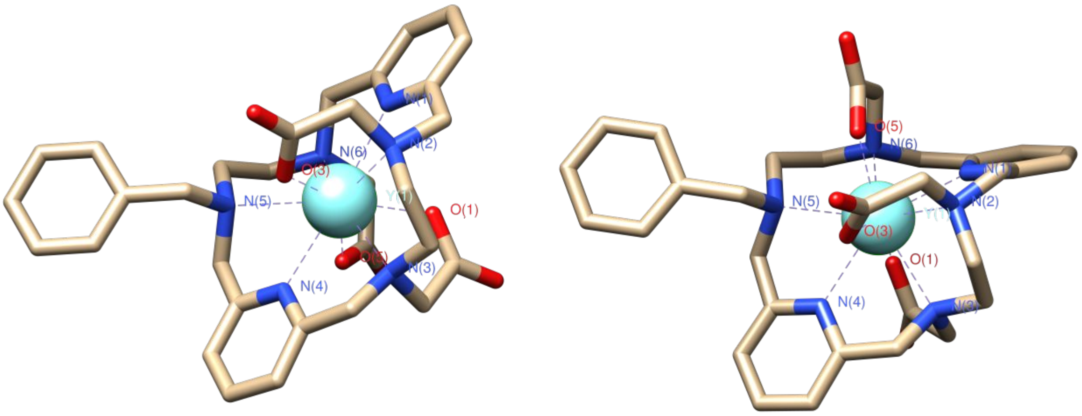

Figure S2. Calculated structures for the *twist-wrap* (left) and *twist-fold* (right) conformations of complex Y**L**^{6}.

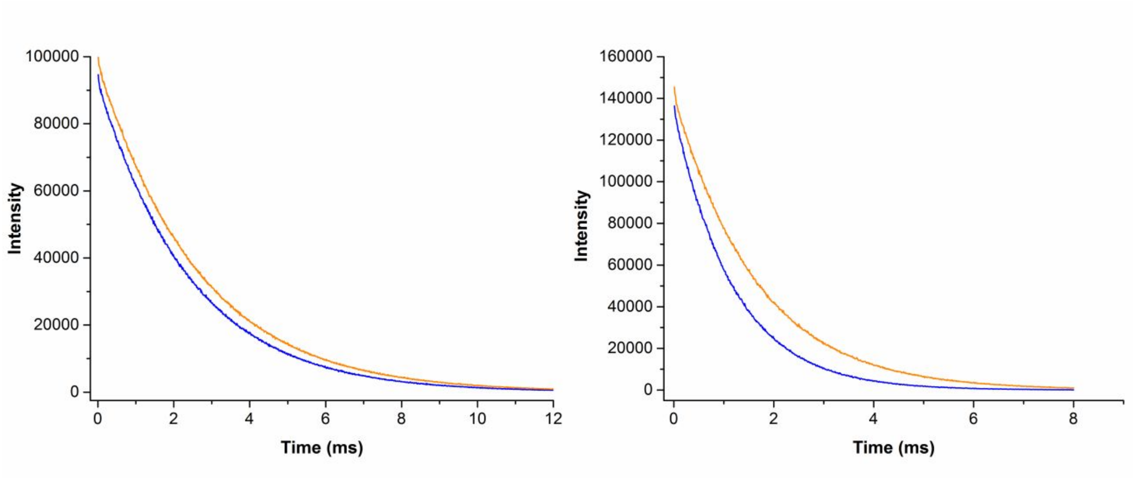

Figure S3. Variation of the emission intensity observed over time in H_2O (blue) and D_2O (orange) solutions for 10^{-5} M Tb$\mathbf{L^6}$ (left) and 10^{-5} M Eu$\mathbf{L^6}$ (right) measured at 542 and 617 nm, respectively.

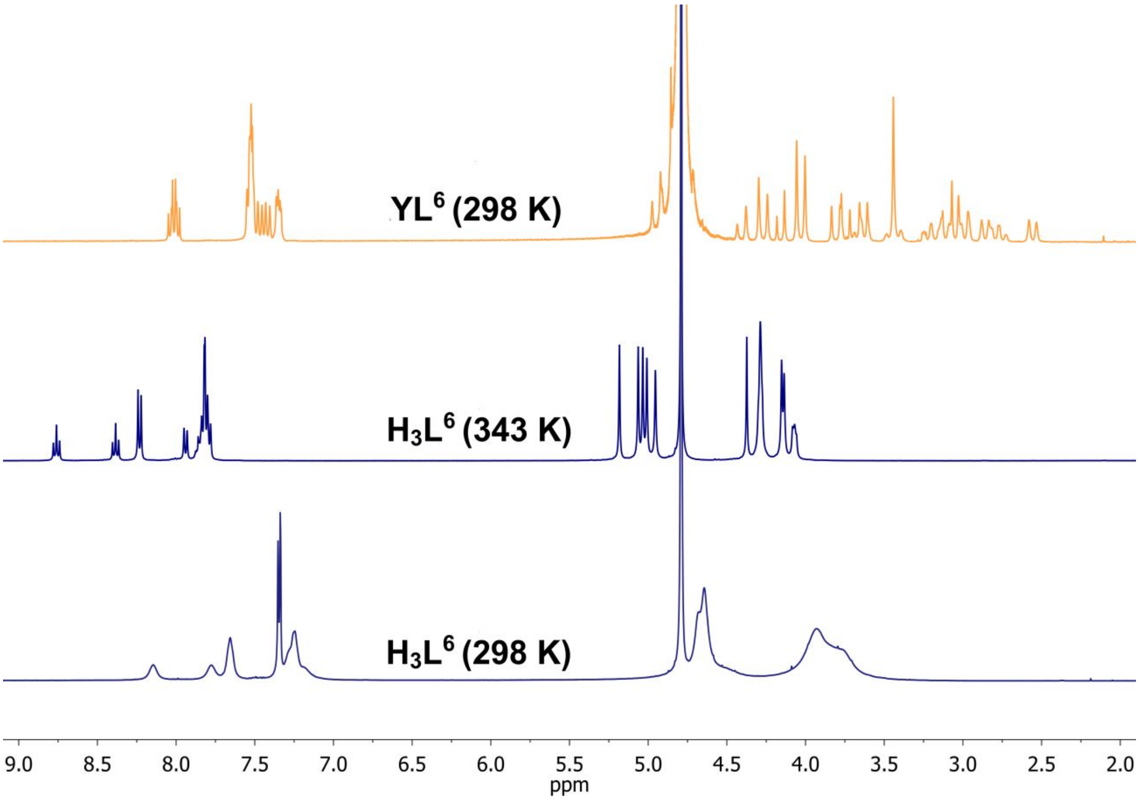

Figure S4. Comparison between the ^{1}H-NMR spectra of ligand $H_3\mathbf{L^6}$ (at 298 and 343 K) and its Y^{3+} complex.

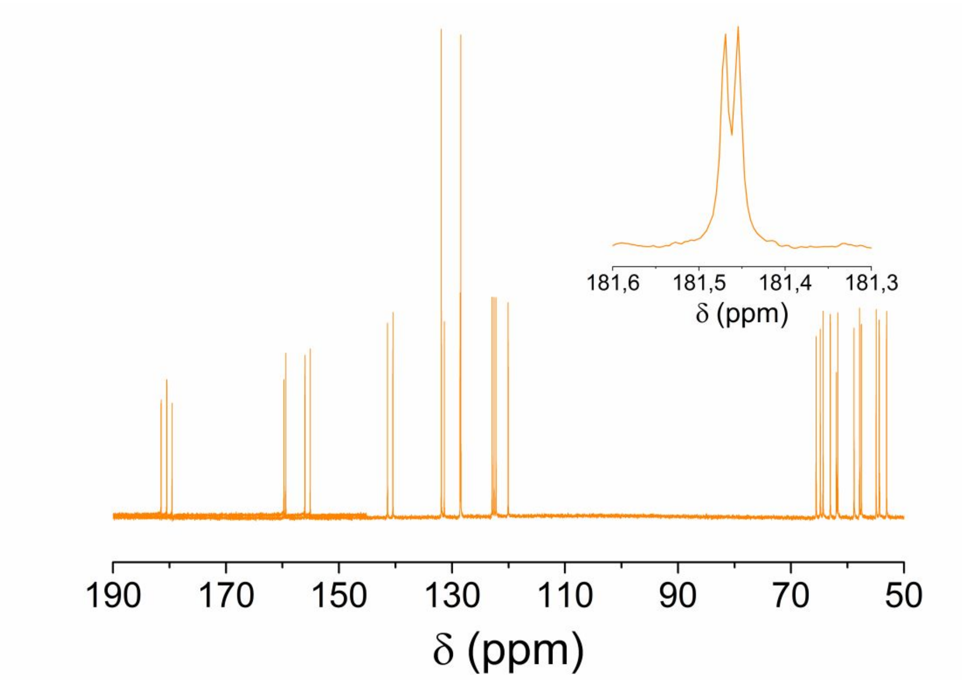

Figure S5. $^{13}C\{^{1}H\}$-NMR spectrum of Y**L⁶** and detail of the splitting of one of the carbonyl signals due to its coupling with the metal center.

Table S1. Bond distances (Å) of the coordination environment of the metal (M) for the *twist-wrap* (tw) and *twist-fold* (tf) conformations of the complexes.

	EuL^6		TbL^6		YL^6		
	**tw**	**tf**	**tw**	**tf**	**tw**	**tf**	**Exp.**
M-N_1	2.667	2.602	2.659	2.585	2.651	2.551	2.517
M-N_2	2.695	2.709	2.684	2.695	2.674	2.674	2.657
M-N_3	2.645	2.701	2.627	2.680	2.599	2.646	2.637
M-N_4	2.624	2.611	2.612	2.591	2.591	2.557	2.519
M-N_5	2.805	2.700	2.815	2.680	2.837	2.650	2.586
M-N_6	2.683	2.663	2.671	2.648	2.659	2.632	2.625
M-O_1	2.407	2.370	2.379	2.343	2.315	2.282	2.302
M-O_3	2.383	2.381	2.343	2.352	2.270	2.292	2.310
M-O_5	2.431	2.370	2.401	2.341	2.334	2.273	2.302

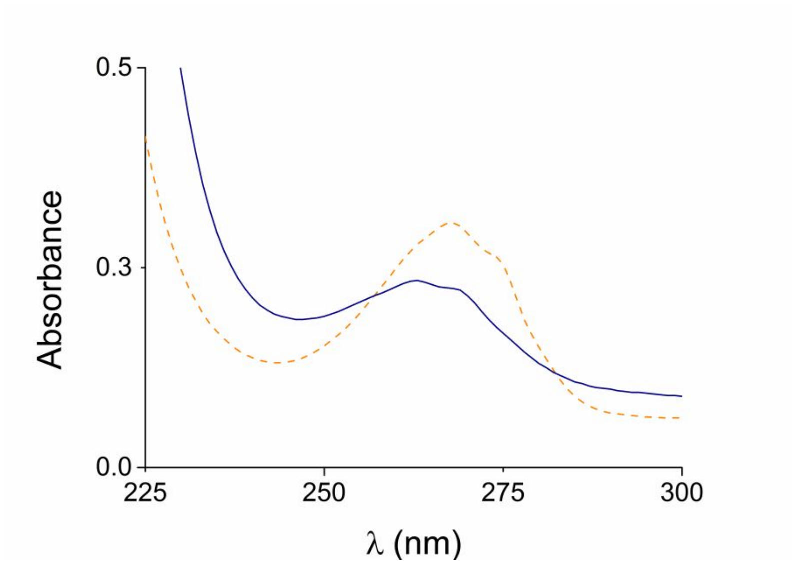

Figure S6. Comparison between UV/Vis absorption spectra of the complex Y**L⁶** (orange, dashed) and the ligand H_3**L⁶** (blue) at pH 7.0 and concentration 2 x 10^{-5} M.

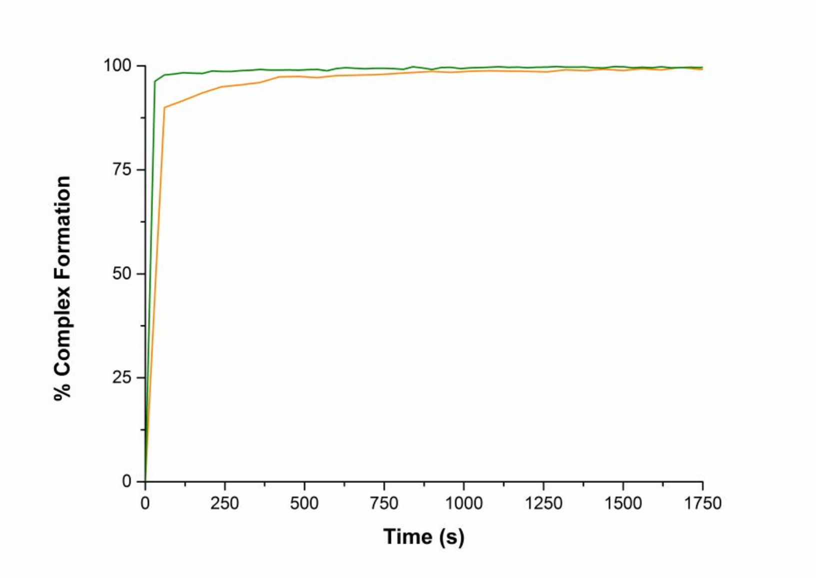

Figure S7. Time course of the complexation of ligand **L⁶** (2 x 10^{-5} M) with Y^{3+} (orange) and Tb^{3+} (green) measured after the addition of 10 equivalents of metal at pH 5.4 in N-methylpiperazine buffered solution

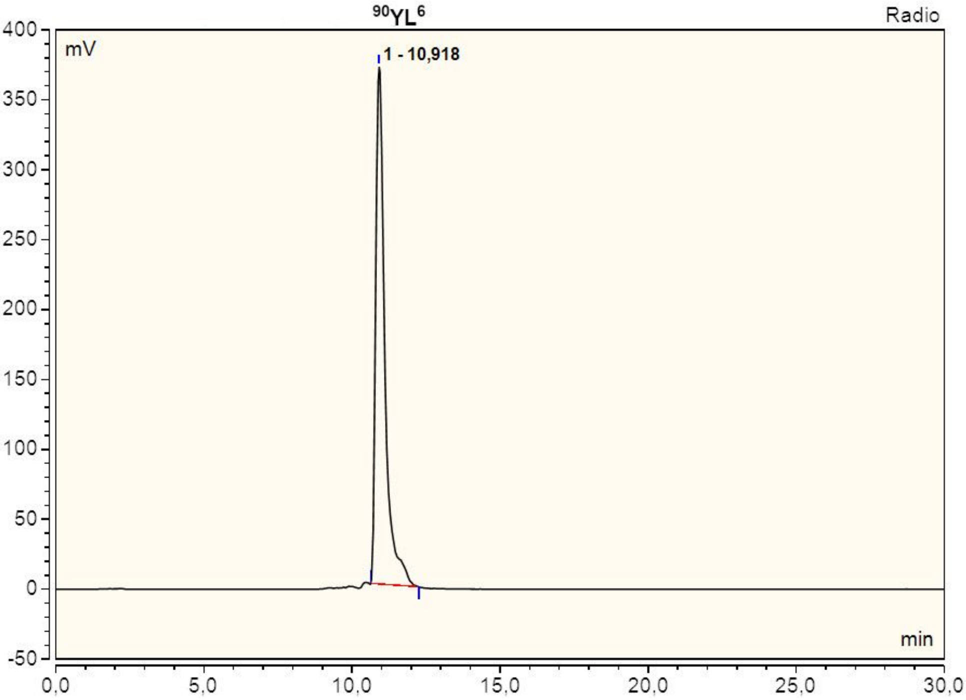

Figure S8. HPLC chromatogram of [^{90}Y]Y**L^{6}**.

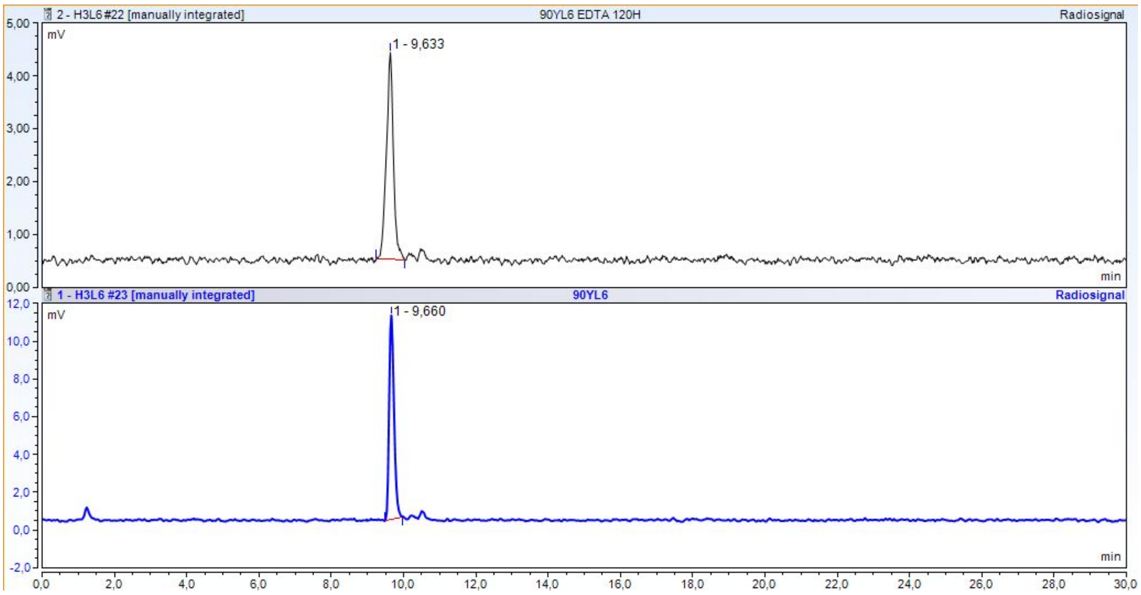

Figure S9. HPLC chromatogram of [^{90}Y]Y**L^{6}** before and after incubation with 100 equivalents of EDTA.

Table S2. MPLC purification method A.

Column volumes (CV)	Time (min:s)	Flow rate (mL/min)	%A	%B
0.00	0	15	100	0
6.00	8:12	15	100	0
8.11	10:43	19.1	59	41
9.95	12:41	19.1	59	41
13.82	16:43	25	0	100
16.95	18:49	25	0	100

Table S3. MPLC purification method B.

Column volumes (CV)	Time (min:s)	Flow rate (mL/min)	%A	%B
0.00	0	15	100	0
6.00	8:12	15	100	0
7.99	10:55	15	65	35
11.47	15:41	15	65	35
15.36	21:07	15	0	100
19.36	26:35	15	0	100

Table S4. Crystal Data and Structure Refinement Details.

Parameter	YL^6	TbL^6
**Formula**	$C_{31}H_{33}N_6O_6Y$	$C_{31}H_{34}N_6O_6Tb$
**Molecular weight, MW**	674.54	745.56
**Crystal system**	orthorhombic	monoclinic
**Space group**	Pbca	$P2_1/n$
a	12.9033(8) Å	9.1899(9) Å
b, β	13.0090(8) Å	38.670(4) Å 90.665(3)°
c	38.405(2) Å	9.1852(9) Å
V	6446.7(7) $Å^3$	3263.9(5) $Å^3$
***F*(000)**	3184	1500
Z	8	4
D_{calc}	1.390 g cm^{-3}	1.517 g cm^{-3}
μ	1.860 mm^{-1}	2.217 mm^{-1}
***θ* range**	2.29 ° –28.36°	2.28° – 28.37°
R_{int}	0.0396	0.0488
**Measured reflections**	152355[a]	69189[b]
**Goodness of fit, GOF on F^2**	1.056	1.019
R_1	0.0378	0.0325
wR_2 **(all data)**	0.1265	0.0629
**Largest differences peak and hole (e $Å^{-3}$)**	0.990 and –0.720	0.749 and –1.835

[a] Of which 8049 were independent and 6957 were unique with $I > 2\sigma(I)$. [b] Of which 8161 were independent and 6986 were unique with $I > 2\sigma(I)$.

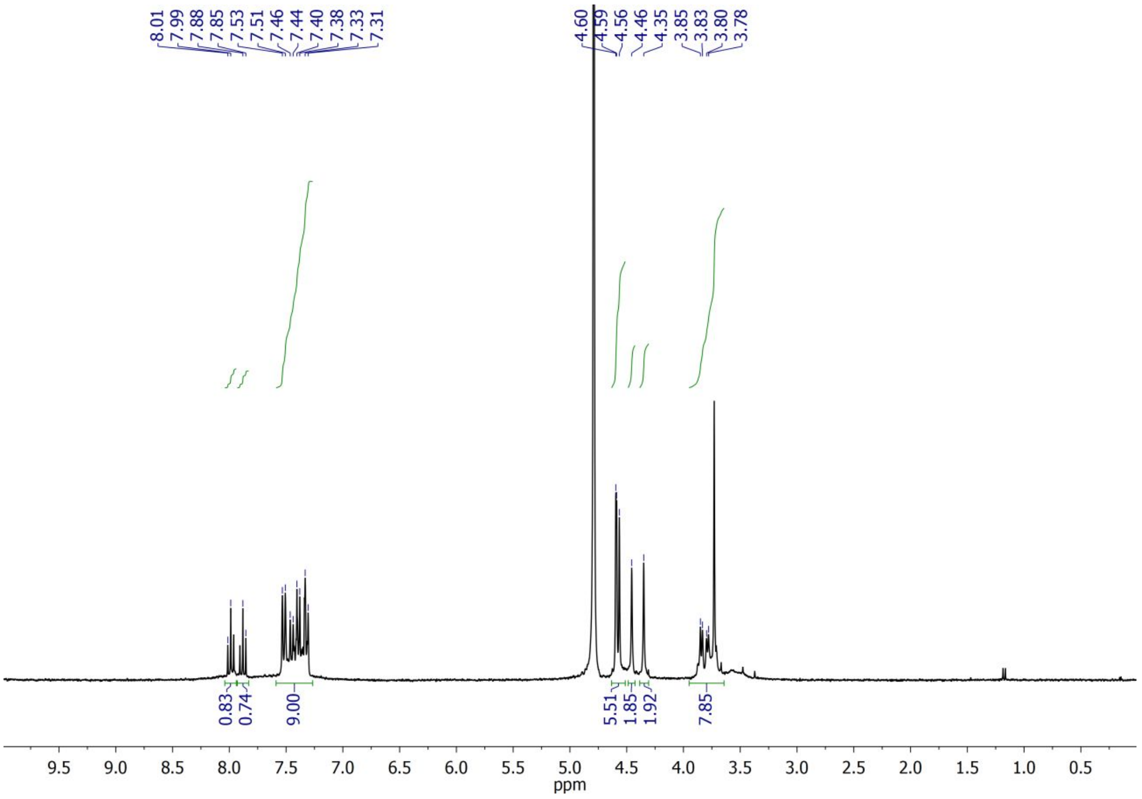

Figure S10. ^{1}H-NMR spectrum of compound **2** (300 MHz, D_2O, 298 K)

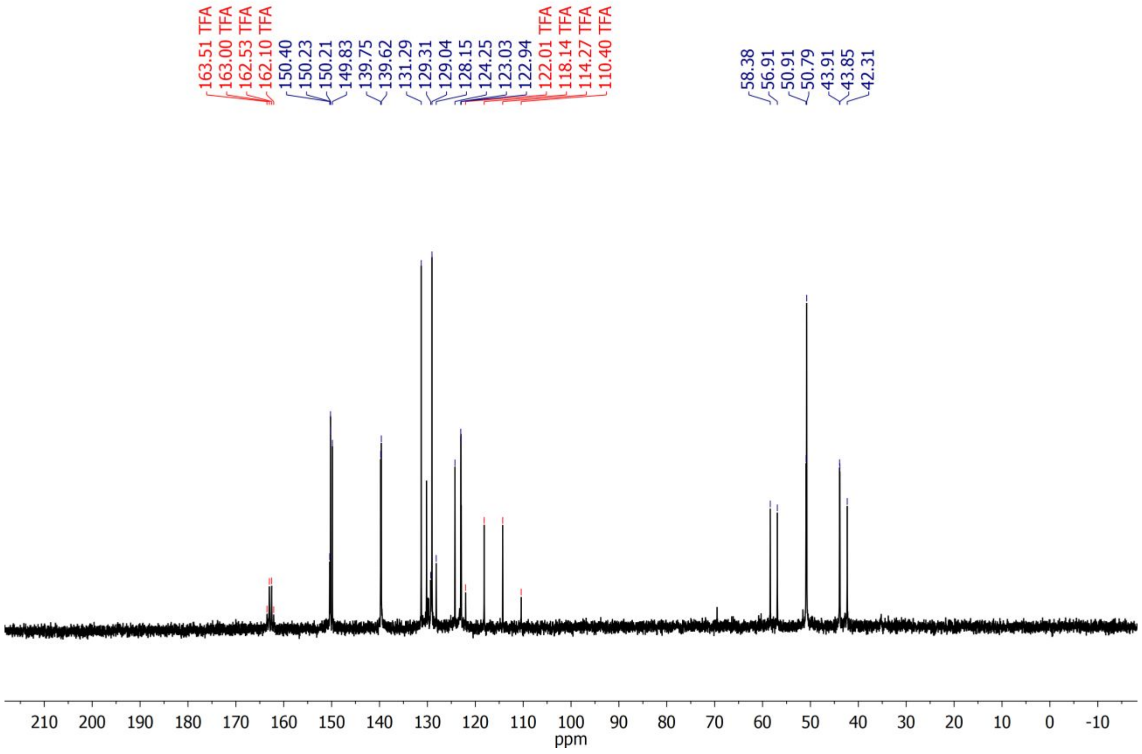

Figure S11. $^{13}C\{^{1}H\}$-NMR spectrum of compound **2** (75 MHz, D_2O, 298 K)

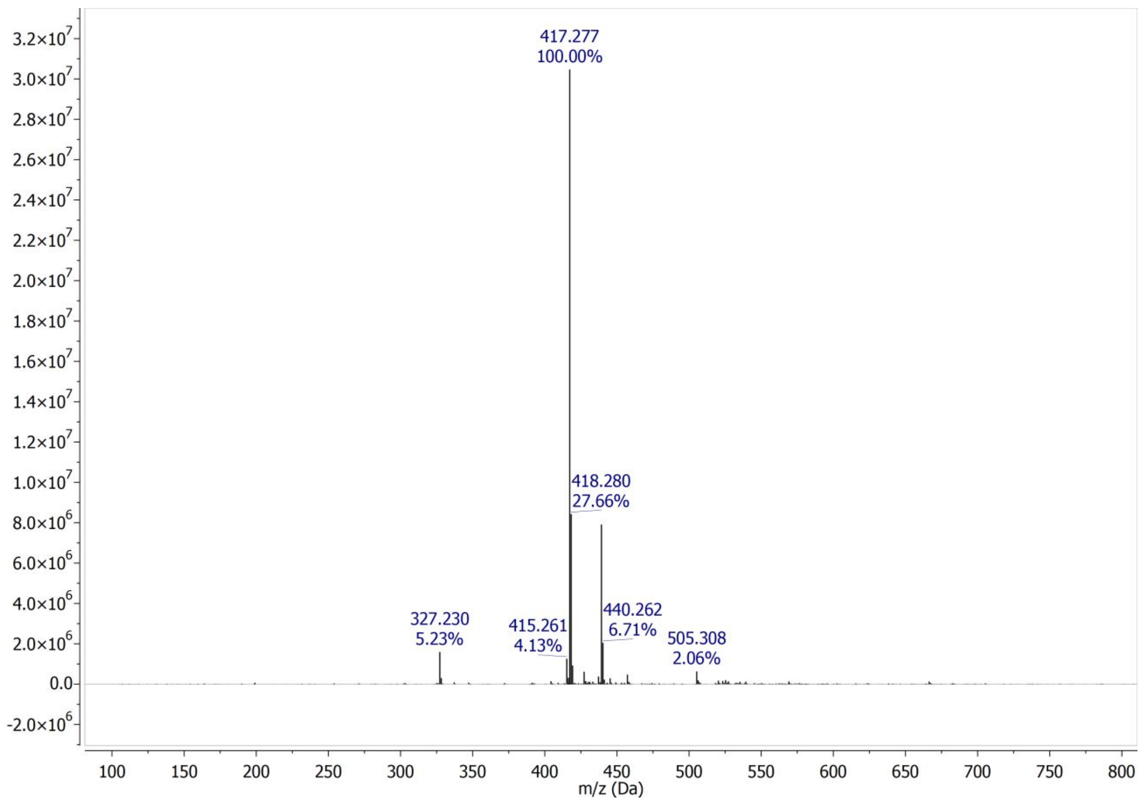

Figure S12. Experimental high resolution mass spectrum (ESI^+) of compound **2**

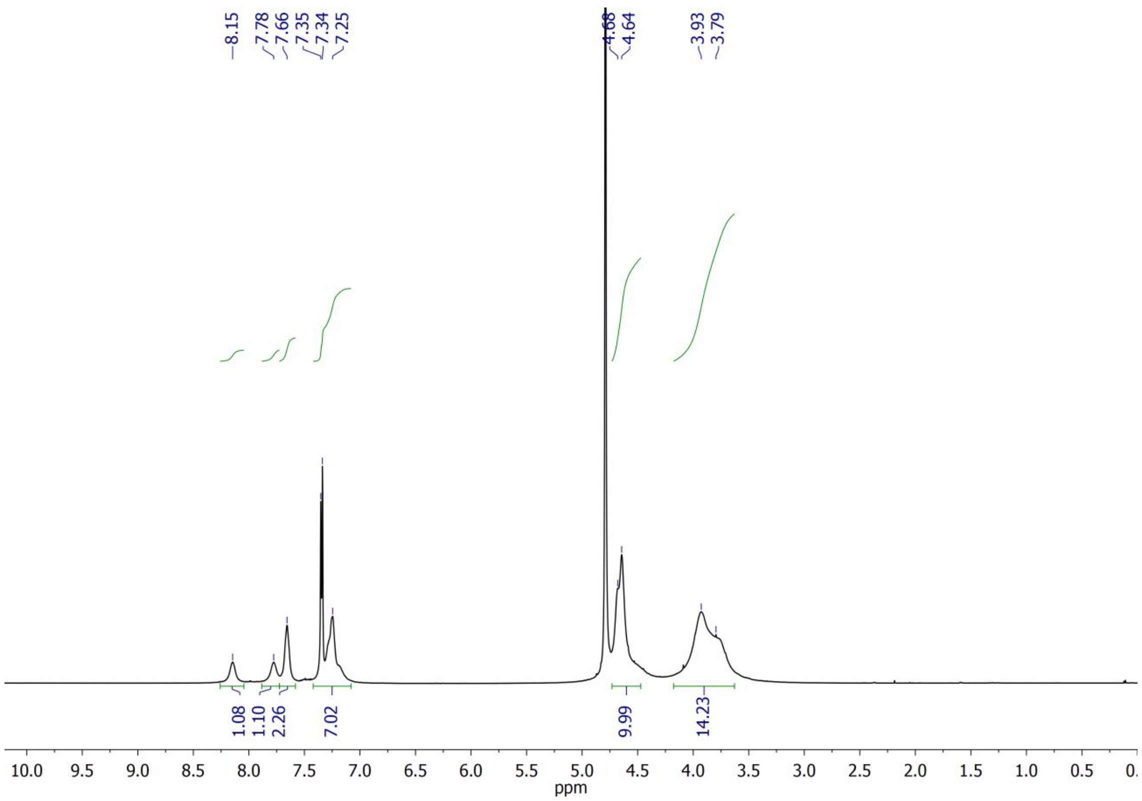

Figure S13. ^{1}H-NMR spectrum of $H_3\mathbf{L^6}$ (500 MHz, D_2O, 298 K, pH ~ 1)

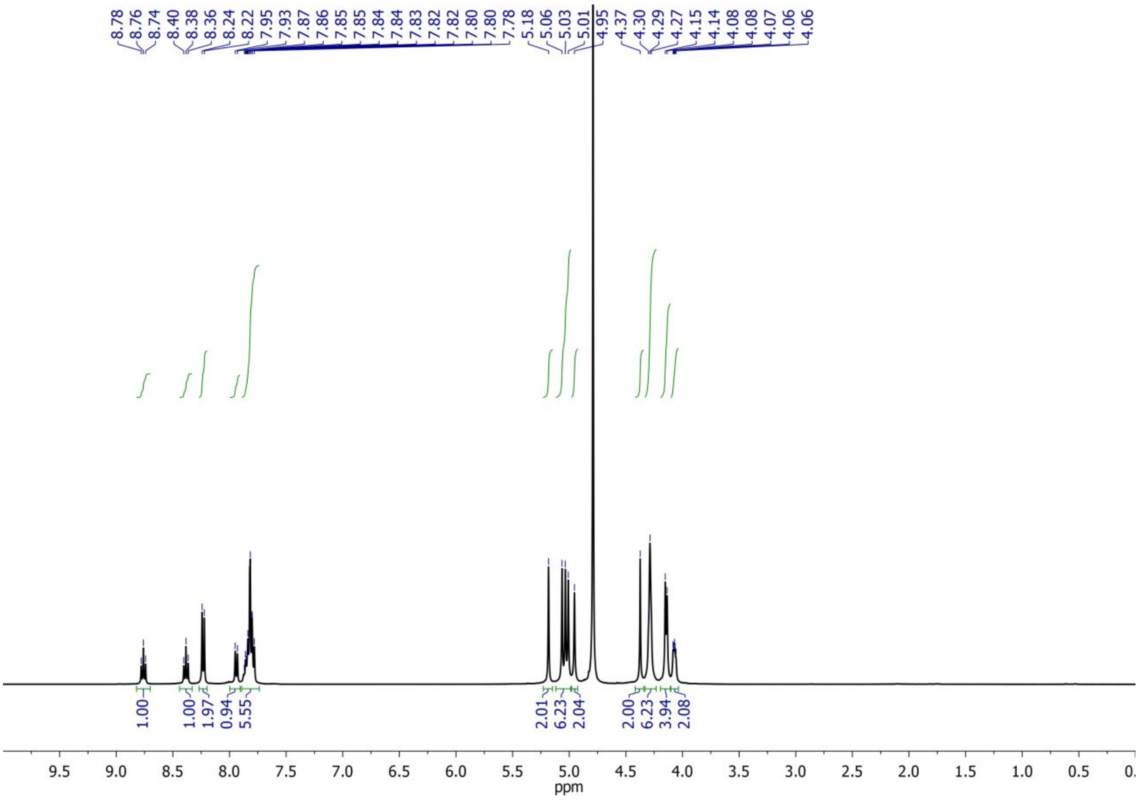

Figure S14. High temperature ^{1}H-NMR spectrum of $H_3\mathbf{L^6}$ (400 MHz, D_2O, 343 K, pH ~ 1).

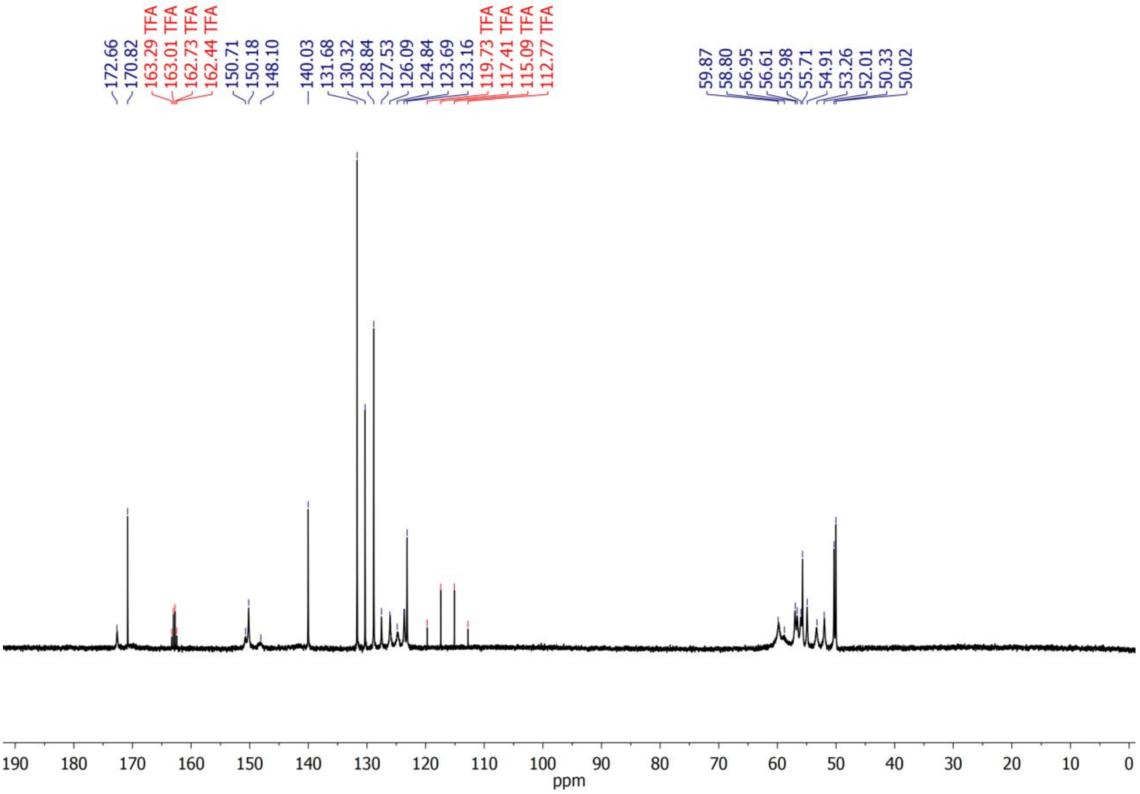

Figure S15. $^{13}C\{^{1}H\}$-NMR spectrum of $H_3\mathbf{L^6}$ (126 MHz, D_2O, 298 K, pH ~1).

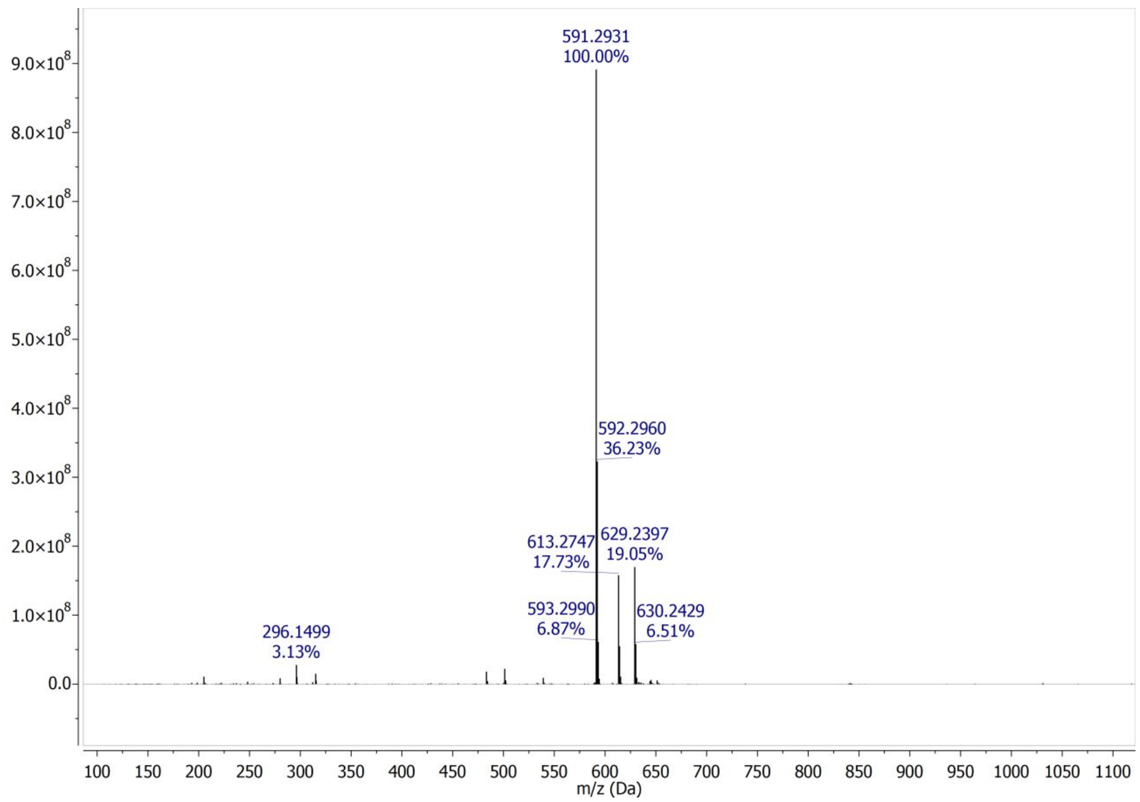

Figure S16. Experimental high resolution mass spectrum (ESI+) of compound **L⁶**

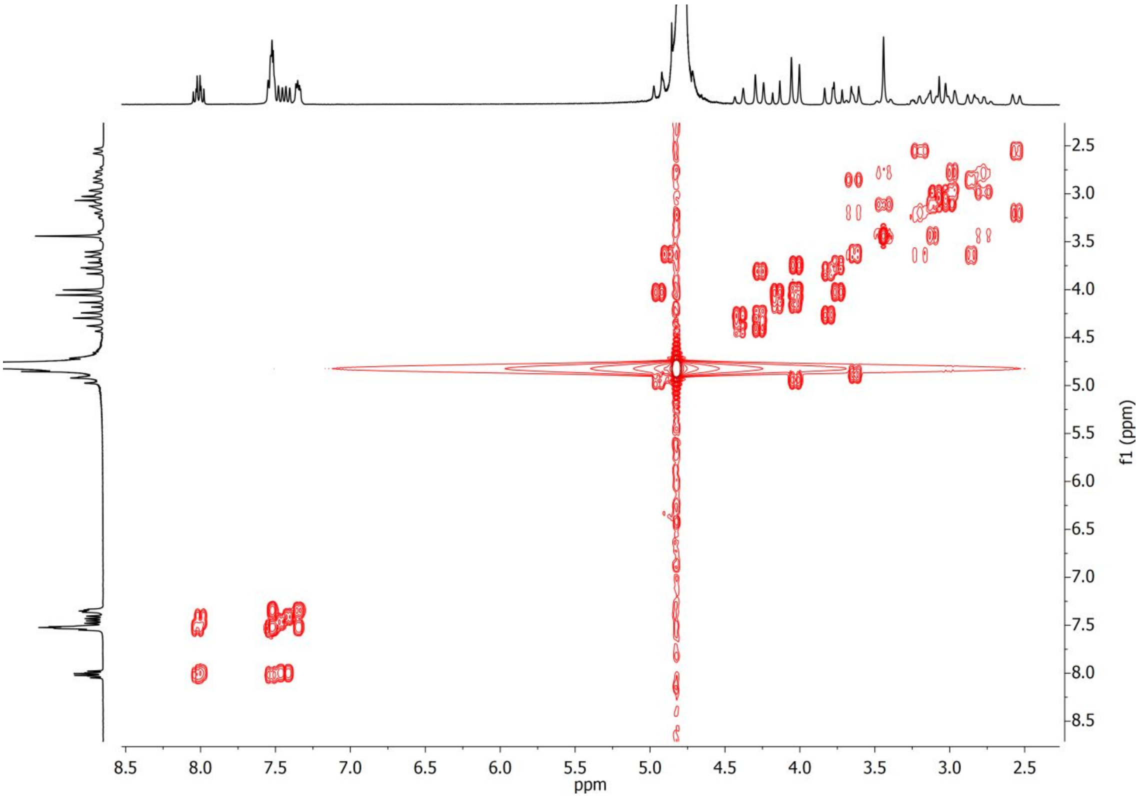

Figure S17. ^{1}H-^{1}H COSY NMR spectrum of Y**L⁶** recorded in D_2O solution (10 mM).

YL[6] *twist-wrap* TPSSh/ECP28MWB/6-31G(d,p), aqueous solution (IEFPCM), 0 imaginary frequencies

Center Number	Atomic Number	Atomic Type	Coordinates (Angstroms) X	Y	Z
1	7	0	1.143607	2.002053	-0.117465
2	7	0	1.993965	-0.660293	-0.429109
3	7	0	-0.456400	-1.834069	-1.736980
4	7	0	-2.400510	-1.835270	0.252156
5	7	0	-2.314642	0.150236	2.116193
6	7	0	-1.487020	2.570104	0.588212
7	8	0	-0.288679	0.775127	-2.222977
8	8	0	0.321835	0.035274	2.025703
9	8	0	-2.740062	0.773744	-0.883903
10	6	0	0.886037	3.244754	0.337419
11	6	0	1.775445	4.308415	0.162070
12	1	0	1.518931	5.295699	0.530943
13	6	0	2.985673	4.071395	-0.486934
14	1	0	3.700208	4.874720	-0.634145
15	6	0	3.261990	2.783250	-0.943601
16	1	0	4.193972	2.556999	-1.450735
17	6	0	2.313928	1.774038	-0.750871
18	6	0	2.541490	0.390584	-1.310286
19	1	0	2.010761	0.345283	-2.264480
20	1	0	3.612190	0.240714	-1.509939
21	6	0	1.969556	-1.970675	-1.116944
22	1	0	1.750061	-2.726347	-0.355064
23	1	0	2.952511	-2.223546	-1.542528
24	6	0	0.933294	-2.036570	-2.228415
25	1	0	1.136478	-1.271787	-2.980225
26	1	0	1.016011	-3.007555	-2.738449
27	6	0	-0.983221	-3.121989	-1.239648
28	1	0	-0.242384	-3.545293	-0.551437
29	1	0	-1.110717	-3.842239	-2.060970
30	6	0	-2.283473	-2.950919	-0.496502
31	6	0	-3.287048	-3.919543	-0.562666
32	1	0	-3.154862	-4.794897	-1.189341
33	6	0	-4.446038	-3.738095	0.191914
34	1	0	-5.240711	-4.476670	0.168935
35	6	0	-4.565026	-2.592983	0.977197
36	1	0	-5.446859	-2.417752	1.584285
37	6	0	-3.526843	-1.657467	0.973059
38	6	0	-3.642884	-0.375453	1.761714
39	1	0	-4.145024	0.361300	1.127675
40	1	0	-4.268104	-0.537915	2.652825
41	6	0	-2.406278	1.527659	2.657438
42	1	0	-1.470492	1.730963	3.186408
43	1	0	-3.218276	1.604800	3.396636
44	6	0	-2.615395	2.558509	1.561570
45	1	0	-3.530644	2.336171	1.009542
46	1	0	-2.744720	3.554567	2.008065
47	6	0	-0.403036	3.434047	1.098710
48	1	0	-0.224589	3.160949	2.145033
49	1	0	-0.698146	4.493122	1.084735
50	6	0	2.797067	-0.763115	0.835307
51	1	0	2.276988	-1.483599	1.469642
52	1	0	2.720749	0.205857	1.330938
53	6	0	-1.283890	-1.311847	-2.849445
54	1	0	-2.330027	-1.287696	-2.525575

55	1	0	-1.211645	-1.947857	-3.742721
56	6	0	-0.865936	0.131157	-3.187505
57	6	0	-1.660108	-0.709175	3.124658
58	1	0	-1.824362	-1.758541	2.857557
59	1	0	-2.076785	-0.553165	4.129183
60	6	0	-0.145274	-0.460899	3.128448
61	6	0	-1.992744	3.041658	-0.722199
62	1	0	-1.143071	3.174207	-1.400117
63	1	0	-2.519092	4.001865	-0.632844
64	6	0	-2.923406	1.975967	-1.332065
65	39	0	-0.685302	0.169624	-0.004488
66	8	0	-1.109312	0.574535	-4.318978
67	8	0	-3.752900	2.318828	-2.185801
68	8	0	0.520205	-0.762630	4.127783
69	6	0	4.256288	-1.157638	0.678108
70	6	0	4.648099	-2.507222	0.689819
71	6	0	5.255007	-0.176694	0.554329
72	6	0	5.992356	-2.866606	0.557879
73	1	0	3.896927	-3.282815	0.818776
74	6	0	6.600903	-0.530799	0.420802
75	1	0	4.976541	0.874295	0.580475
76	6	0	6.972470	-1.878617	0.417566
77	1	0	6.274819	-3.915376	0.573439
78	1	0	7.356793	0.243836	0.328675
79	1	0	8.017635	-2.156761	0.318621

E(RTPSSh) = -2021.0698237 Hartree

Zero-point correction = 0.641075
Thermal correction to Energy = 0.678980
Thermal correction to Enthalpy = 0.679924
Thermal correction to Gibbs Free Energy = 0.573568
Sum of electronic and zero-point Energies = -2020.428748
Sum of electronic and thermal Energies = -2020.390844
Sum of electronic and thermal Enthalpies = -2020.389899
Sum of electronic and thermal Free Energies = -2020.496256

YL[6] *twist-fold* TPSSh/ECP28MWB/6-31G(d,p), aqueous solution (IEFPCM), 0 imaginary frequencies

Center Number	Atomic Number	Atomic Type	Coordinates (Angstroms) X	Y	Z
1	6	0	-2.923752	-1.621197	-1.689483
2	6	0	-4.187562	-1.919553	-2.205665
3	1	0	-4.285897	-2.290158	-3.220469
4	6	0	-5.305745	-1.742671	-1.390903
5	1	0	-6.300874	-1.948001	-1.771987
6	6	0	-5.126359	-1.341197	-0.065666
7	1	0	-5.968771	-1.247254	0.611199
8	6	0	-3.830304	-1.085781	0.387426
9	6	0	-3.532279	-0.870707	1.855590
10	1	0	-4.429803	-0.516931	2.379476
11	1	0	-3.298861	-1.860676	2.263640
12	6	0	-2.812694	1.407832	2.364850
13	1	0	-2.023014	1.907698	2.929420
14	1	0	-3.711739	1.415871	2.998221
15	6	0	-3.097778	2.166930	1.078753
16	1	0	-3.852230	1.633763	0.493480
17	1	0	-3.511853	3.157094	1.322648
18	6	0	-0.949732	3.279026	0.839312
19	1	0	-0.789564	3.004897	1.885231
20	1	0	-1.389546	4.286574	0.812684
21	6	0	0.395192	3.284816	0.155844
22	6	0	1.123353	4.462175	-0.035387
23	1	0	0.716740	5.413389	0.290872
24	6	0	2.372570	4.384638	-0.652053
25	1	0	2.967649	5.279510	-0.802524
26	6	0	2.836187	3.145306	-1.095040
27	1	0	3.789348	3.052077	-1.604603
28	6	0	2.037353	2.016965	-0.889540
29	7	0	1.929750	-0.455291	-0.637835
30	6	0	2.389358	0.673071	-1.478738
31	6	0	1.970870	-1.713487	-1.432521
32	1	0	2.893913	-1.774826	-2.025399
33	1	0	1.990364	-2.550034	-0.729416
34	6	0	2.760573	-0.573332	0.613432
35	1	0	2.295910	-1.369464	1.200067
36	1	0	2.621820	0.360950	1.163542
37	6	0	0.766494	-1.847692	-2.361837
38	1	0	0.921296	-2.721564	-3.014127
39	1	0	0.671592	-0.967013	-3.002729
40	6	0	-1.662530	-1.831399	-2.499271
41	1	0	-1.786610	-2.711315	-3.148164
42	1	0	-1.489269	-0.956459	-3.130706
43	6	0	-1.584470	-0.451343	3.283040
44	1	0	-1.436169	-1.530386	3.192403
45	1	0	-2.099522	-0.247332	4.231894
46	6	0	-0.182993	0.185464	3.302378
47	6	0	-2.233192	2.702866	-1.148650
48	1	0	-2.181647	3.791615	-1.265840
49	1	0	-3.269059	2.405261	-1.342717
50	6	0	-1.387712	2.018559	-2.237705
51	6	0	-0.557306	-3.268931	-0.874232
52	1	0	0.026473	-4.040619	-1.392251
53	1	0	-1.595410	-3.615642	-0.859673
54	6	0	-0.117400	-3.188020	0.595699

55	7	0	-2.759611	-1.166757	-0.428868
56	7	0	-2.369593	0.010749	2.113810
57	7	0	-1.879862	2.288495	0.234944
58	7	0	0.859427	2.086293	-0.243164
59	7	0	-0.496651	-1.974980	-1.599643
60	8	0	0.216590	0.698235	2.181383
61	8	0	-0.941989	0.840893	-1.941469
62	8	0	-0.089678	-2.004024	1.116543
63	1	0	1.870764	0.601183	-2.440638
64	1	0	3.466513	0.609998	-1.683044
65	39	0	-0.562175	-0.004616	0.143610
66	6	0	4.238841	-0.850256	0.409552
67	6	0	5.160630	0.206672	0.318672
68	6	0	4.723206	-2.166815	0.327223
69	6	0	6.522567	-0.041874	0.124048
70	1	0	4.810171	1.231777	0.415410
71	6	0	6.083877	-2.419809	0.133122
72	1	0	4.033055	-3.000384	0.430659
73	6	0	6.986677	-1.357039	0.024948
74	1	0	7.219032	0.788879	0.057349
75	1	0	6.439216	-3.444562	0.075286
76	1	0	8.044569	-1.552854	-0.123196
77	8	0	0.139915	-4.245144	1.189762
78	8	0	-1.252795	2.590434	-3.328517
79	8	0	0.468757	0.145370	4.355460

E(RTPSSh) = -2021.0753222 Hartree

Zero-point correction = 0.641098
Thermal correction to Energy = 0.679076
Thermal correction to Enthalpy = 0.680021
Thermal correction to Gibbs Free Energy = 0.572473
Sum of electronic and zero-point Energies = -2020.434225
Sum of electronic and thermal Energies = -2020.396246
Sum of electronic and thermal Enthalpies = -2020.395302
Sum of electronic and thermal Free Energies = -2020.502849

EuL⁶ *twist-wrap* TPSSh/LCRECP/6-31G(d,p), aqueous solution (IEFPCM), 0 imaginary frequencies

Center Number	Atomic Number	Atomic Type	Coordinates (Angstroms) X	Y	Z
1	7	0	1.210696	2.012345	-0.138606
2	7	0	2.036184	-0.664230	-0.440485
3	7	0	-0.416052	-1.885949	-1.749565
4	7	0	-2.359469	-1.868463	0.258053
5	7	0	-2.288827	0.166036	2.117794
6	7	0	-1.436318	2.587912	0.552017
7	8	0	-0.229216	0.725858	-2.320362
8	8	0	0.370419	0.152122	2.175499
9	8	0	-2.729090	0.801937	-0.933460
10	6	0	0.947768	3.254793	0.313369
11	6	0	1.842911	4.313735	0.138861
12	1	0	1.591373	5.303777	0.503449
13	6	0	3.055140	4.068190	-0.504414
14	1	0	3.773220	4.868776	-0.649505
15	6	0	3.331916	2.779093	-0.959717
16	1	0	4.265118	2.552146	-1.464124
17	6	0	2.379953	1.773528	-0.768521
18	6	0	2.592113	0.383972	-1.325078
19	1	0	2.052443	0.339611	-2.274741
20	1	0	3.659692	0.220914	-1.527894
21	6	0	2.014442	-1.982764	-1.121604
22	1	0	1.801085	-2.732065	-0.351865
23	1	0	2.999363	-2.232000	-1.543119
24	6	0	0.978589	-2.074266	-2.233699
25	1	0	1.171629	-1.314035	-2.992365
26	1	0	1.078491	-3.049600	-2.731949
27	6	0	-0.939071	-3.167710	-1.229524
28	1	0	-0.192864	-3.580601	-0.540747
29	1	0	-1.071507	-3.899875	-2.039304
30	6	0	-2.236913	-2.993485	-0.476173
31	6	0	-3.234113	-3.970205	-0.514258
32	1	0	-3.104611	-4.854807	-1.128220
33	6	0	-4.384766	-3.784654	0.252306
34	1	0	-5.173077	-4.530410	0.250856
35	6	0	-4.507584	-2.627677	1.019905
36	1	0	-5.385967	-2.451088	1.631351
37	6	0	-3.479070	-1.681980	0.988265
38	6	0	-3.606577	-0.376502	1.741327
39	1	0	-4.087560	0.343008	1.071563
40	1	0	-4.255905	-0.513216	2.619000
41	6	0	-2.397797	1.558823	2.623123
42	1	0	-1.478910	1.773245	3.175713
43	1	0	-3.230830	1.648152	3.336876
44	6	0	-2.579410	2.578471	1.509149
45	1	0	-3.484004	2.352550	0.941723
46	1	0	-2.717509	3.577019	1.947770
47	6	0	-0.347426	3.443350	1.070568
48	1	0	-0.173649	3.161328	2.115208
49	1	0	-0.636056	4.504291	1.061966
50	6	0	2.828224	-0.766474	0.833040
51	1	0	2.298029	-1.482575	1.464655
52	1	0	2.753253	0.204796	1.324538
53	6	0	-1.250994	-1.379362	-2.864825
54	1	0	-2.293602	-1.350936	-2.529711

55	1	0	-1.189710	-2.032857	-3.746123
56	6	0	-0.843331	0.058954	-3.245473
57	6	0	-1.651241	-0.662647	3.164176
58	1	0	-1.771138	-1.718711	2.898222
59	1	0	-2.120075	-0.513387	4.146493
60	6	0	-0.143209	-0.367440	3.246844
61	6	0	-1.920736	3.054925	-0.768364
62	1	0	-1.059529	3.165060	-1.436202
63	1	0	-2.428301	4.026528	-0.695165
64	6	0	-2.868346	2.007435	-1.389102
65	8	0	-1.136857	0.472388	-4.376263
66	8	0	-3.667806	2.373650	-2.261109
67	8	0	0.469356	-0.662316	4.281451
68	6	0	4.284570	-1.174243	0.686867
69	6	0	4.664133	-2.527098	0.712299
70	6	0	5.291447	-0.203147	0.554158
71	6	0	6.005112	-2.899581	0.584263
72	1	0	3.906140	-3.294708	0.847862
73	6	0	6.634127	-0.570598	0.424607
74	1	0	5.022436	0.850349	0.570292
75	6	0	6.993780	-1.921623	0.434592
76	1	0	6.278096	-3.950655	0.609283
77	1	0	7.396867	0.196260	0.324788
78	1	0	8.036355	-2.210028	0.337846
79	63	0	-0.615692	0.133622	0.005862

E(RTPSSh) = -2018.0413189 Hartree

Zero-point correction = 0.640426
Thermal correction to Energy = 0.678673
Thermal correction to Enthalpy = 0.679617
Thermal correction to Gibbs Free Energy = 0.572181
Sum of electronic and zero-point Energies = -2017.400893
Sum of electronic and thermal Energies = -2017.362646
Sum of electronic and thermal Enthalpies = -2017.361702
Sum of electronic and thermal Free Energies = -2017.469138

EuL[6] *twist-fold* TPSSh/LCRECP/6-31G(d,p), aqueous solution (IEFPCM), 0 imaginary frequencies

Center Number	Atomic Number	Atomic Type	Coordinates (Angstroms) X	Y	Z
1	6	0	-2.850350	-1.770956	-1.706253
2	6	0	-4.085463	-2.130034	-2.251407
3	1	0	-4.136344	-2.553865	-3.248626
4	6	0	-5.238566	-1.933468	-1.491780
5	1	0	-6.212906	-2.184448	-1.898390
6	6	0	-5.122970	-1.441470	-0.191104
7	1	0	-5.996161	-1.315943	0.440059
8	6	0	-3.851594	-1.134654	0.300675
9	6	0	-3.641102	-0.809424	1.765486
10	1	0	-4.559912	-0.386527	2.190711
11	1	0	-3.481274	-1.770399	2.267447
12	6	0	-2.889828	1.468557	2.266081
13	1	0	-2.112050	1.958936	2.854668
14	1	0	-3.811874	1.503813	2.864151
15	6	0	-3.114514	2.221304	0.962210
16	1	0	-3.844114	1.684252	0.348800
17	1	0	-3.540212	3.211286	1.186129
18	6	0	-0.937673	3.312204	0.800445
19	1	0	-0.787444	3.006209	1.839452
20	1	0	-1.368661	4.323753	0.795992
21	6	0	0.414942	3.325867	0.127181
22	6	0	1.137747	4.506078	-0.065081
23	1	0	0.723766	5.456836	0.252665
24	6	0	2.392000	4.432036	-0.671965
25	1	0	2.982107	5.330077	-0.823059
26	6	0	2.869031	3.194073	-1.104556
27	1	0	3.826542	3.106931	-1.606774
28	6	0	2.078068	2.060315	-0.898643
29	7	0	2.009227	-0.419117	-0.631321
30	6	0	2.447872	0.714094	-1.477932
31	6	0	2.069716	-1.684802	-1.415434
32	1	0	2.987176	-1.728882	-2.018118
33	1	0	2.119710	-2.514591	-0.706114
34	6	0	2.831636	-0.513652	0.626617
35	1	0	2.360712	-1.294859	1.228511
36	1	0	2.697439	0.434784	1.154270
37	6	0	0.861538	-1.861465	-2.334672
38	1	0	1.044432	-2.727959	-2.988967
39	1	0	0.733730	-0.983860	-2.974545
40	6	0	-1.563166	-1.946598	-2.484493
41	1	0	-1.641971	-2.832793	-3.131143
42	1	0	-1.415705	-1.067729	-3.118298
43	6	0	-1.748364	-0.408227	3.268157
44	1	0	-1.602972	-1.488941	3.185168
45	1	0	-2.318635	-0.207283	4.185247
46	6	0	-0.344126	0.217842	3.386980
47	6	0	-2.166073	2.764356	-1.231973
48	1	0	-2.084289	3.851551	-1.345194
49	1	0	-3.202194	2.492362	-1.459209
50	6	0	-1.301549	2.062141	-2.297251
51	6	0	-0.410988	-3.326475	-0.838495
52	1	0	0.139142	-4.100296	-1.388999
53	1	0	-1.449051	-3.665203	-0.756383

54	6	0	0.119763	-3.236934	0.602782
55	7	0	-2.746145	-1.254312	-0.462704
56	7	0	-2.468659	0.055822	2.054664
57	7	0	-1.867121	2.342175	0.162595
58	7	0	0.893762	2.128674	-0.262446
59	7	0	-0.399670	-2.036436	-1.573403
60	8	0	0.134899	0.745223	2.303306
61	8	0	-0.953698	0.844915	-2.020679
62	8	0	0.019603	-2.075359	1.168561
63	1	0	1.929545	0.626739	-2.438859
64	1	0	3.525424	0.667012	-1.684353
65	6	0	4.308990	-0.804980	0.438410
66	6	0	5.239661	0.239412	0.305545
67	6	0	4.781486	-2.127994	0.414687
68	6	0	6.599894	-0.029741	0.126322
69	1	0	4.899485	1.270948	0.360609
70	6	0	6.140285	-2.401254	0.236255
71	1	0	4.083277	-2.949545	0.554017
72	6	0	7.052629	-1.351938	0.085568
73	1	0	7.304081	0.791167	0.027048
74	1	0	6.486635	-3.430597	0.223940
75	1	0	8.109241	-1.562878	-0.050347
76	8	0	0.555501	-4.267750	1.133891
77	8	0	-1.063265	2.662622	-3.353409
78	8	0	0.233320	0.145131	4.479665
79	63	0	-0.543189	-0.011607	0.150189

E(RTPSSh) = -2018.0423173 Hartree

Zero-point correction = 0.640529
Thermal correction to Energy = 0.678913
Thermal correction to Enthalpy = 0.679857
Thermal correction to Gibbs Free Energy = 0.570370
Sum of electronic and zero-point Energies = -2017.401789
Sum of electronic and thermal Energies = -2017.363404
Sum of electronic and thermal Enthalpies = -2017.362460
Sum of electronic and thermal Free Energies = -2017.471947

TbL⁶ *twist-wrap* TPSSh/LCRECP/6-31G(d,p), aqueous solution (IEFPCM), 0 imaginary frequencies

Center Number	Atomic Number	Atomic Type	Coordinates (Angstroms) X	Y	Z
1	7	0	1.199431	2.008130	-0.127703
2	7	0	2.039169	-0.664558	-0.436838
3	7	0	-0.409361	-1.866346	-1.746857
4	7	0	-2.348082	-1.865547	0.256259
5	7	0	-2.275498	0.151478	2.117119
6	7	0	-1.442850	2.573505	0.562232
7	8	0	-0.234864	0.747445	-2.288527
8	8	0	0.381973	0.079154	2.118394
9	8	0	-2.721120	0.778927	-0.918606
10	6	0	0.936286	3.250134	0.325323
11	6	0	1.830617	4.310708	0.156758
12	1	0	1.575474	5.299265	0.522922
13	6	0	3.045319	4.068873	-0.482457
14	1	0	3.763186	4.870395	-0.623269
15	6	0	3.323427	2.780962	-0.939695
16	1	0	4.258016	2.555051	-1.442000
17	6	0	2.371690	1.774085	-0.754529
18	6	0	2.589520	0.388918	-1.317021
19	1	0	2.050690	0.347511	-2.267106
20	1	0	3.657920	0.232205	-1.521290
21	6	0	2.019792	-1.976888	-1.126797
22	1	0	1.807590	-2.733001	-0.363421
23	1	0	3.004296	-2.222253	-1.551958
24	6	0	0.982628	-2.055871	-2.237653
25	1	0	1.176815	-1.290687	-2.991134
26	1	0	1.076435	-3.027336	-2.744497
27	6	0	-0.928407	-3.153686	-1.237635
28	1	0	-0.180664	-3.570994	-0.553290
29	1	0	-1.059957	-3.878789	-2.053856
30	6	0	-2.224745	-2.987045	-0.482800
31	6	0	-3.219402	-3.966087	-0.526188
32	1	0	-3.086661	-4.847229	-1.144440
33	6	0	-4.371054	-3.787239	0.240119
34	1	0	-5.157780	-4.534640	0.234771
35	6	0	-4.495919	-2.633963	1.012724
36	1	0	-5.374511	-2.461242	1.624998
37	6	0	-3.468887	-1.686640	0.986216
38	6	0	-3.596118	-0.388175	1.748813
39	1	0	-4.085263	0.334958	1.089290
40	1	0	-4.236947	-0.533155	2.631397
41	6	0	-2.383076	1.539093	2.634348
42	1	0	-1.458411	1.753852	3.177816
43	1	0	-3.207667	1.621348	3.358583
44	6	0	-2.580576	2.560290	1.525484
45	1	0	-3.488312	2.331018	0.964362
46	1	0	-2.719762	3.558052	1.965130
47	6	0	-0.360776	3.439100	1.076867
48	1	0	-0.189285	3.169589	2.125182
49	1	0	-0.655547	4.498149	1.057484
50	6	0	2.837762	-0.770992	0.831457
51	1	0	2.314312	-1.493135	1.461309
52	1	0	2.759760	0.196518	1.329536
53	6	0	-1.246494	-1.353844	-2.857773
54	1	0	-2.288362	-1.326962	-2.520587

55	1	0	-1.185434	-2.000874	-3.743763
56	6	0	-0.837967	0.086359	-3.226112
57	6	0	-1.632251	-0.686290	3.152618
58	1	0	-1.767509	-1.740421	2.886923
59	1	0	-2.084485	-0.533041	4.141904
60	6	0	-0.121603	-0.408129	3.210468
61	6	0	-1.935472	3.038486	-0.756183
62	1	0	-1.077401	3.154931	-1.426726
63	1	0	-2.450319	4.005887	-0.679744
64	6	0	-2.876410	1.983775	-1.372743
65	8	0	-1.118229	0.508094	-4.356714
66	8	0	-3.685146	2.341515	-2.239144
67	8	0	0.504362	-0.684913	4.241297
68	6	0	4.296062	-1.169442	0.677438
69	6	0	4.683222	-2.520318	0.691417
70	6	0	5.297955	-0.191999	0.553327
71	6	0	6.026301	-2.884508	0.561171
72	1	0	3.929409	-3.293150	0.820556
73	6	0	6.642737	-0.550981	0.421522
74	1	0	5.023309	0.859884	0.578621
75	6	0	7.009833	-1.900032	0.420594
76	1	0	6.305018	-3.934257	0.577898
77	1	0	7.401338	0.220902	0.328865
78	1	0	8.054098	-2.181866	0.322615
79	65	0	-0.622197	0.141279	0.002298

E(RTPSSh) = -2019.2194126 Hartree

Zero-point correction = 0.640774
Thermal correction to Energy = 0.678943
Thermal correction to Enthalpy = 0.679887
Thermal correction to Gibbs Free Energy = 0.572558
Sum of electronic and zero-point Energies = -2018.578639
Sum of electronic and thermal Energies = -2018.540469
Sum of electronic and thermal Enthalpies = -2018.539525
Sum of electronic and thermal Free Energies = -2018.646855

TbL^6 *twist-fold* TPSSh/LCRECP/6-31G(d,p), aqueous solution (IEFPCM), 0 imaginary frequencies

Center Number	Atomic Number	Atomic Type	Coordinates (Angstroms) X	Y	Z
1	6	0	-2.855408	-1.731220	-1.703305
2	6	0	-4.099695	-2.075818	-2.237198
3	1	0	-4.166234	-2.481616	-3.240982
4	6	0	-5.240757	-1.891452	-1.456752
5	1	0	-6.221840	-2.131314	-1.853744
6	6	0	-5.103648	-1.430236	-0.146598
7	1	0	-5.965894	-1.320074	0.502208
8	6	0	-3.824389	-1.134875	0.330423
9	6	0	-3.581001	-0.849695	1.797597
10	1	0	-4.494418	-0.457491	2.262418
11	1	0	-3.386408	-1.821704	2.264802
12	6	0	-2.854132	1.432644	2.298029
13	1	0	-2.074958	1.931847	2.877328
14	1	0	-3.767653	1.452448	2.909704
15	6	0	-3.106356	2.184695	0.999872
16	1	0	-3.841223	1.643271	0.396864
17	1	0	-3.534899	3.171882	1.230076
18	6	0	-0.946219	3.296040	0.811149
19	1	0	-0.789671	3.000708	1.852007
20	1	0	-1.386507	4.303481	0.800204
21	6	0	0.403239	3.315574	0.134031
22	6	0	1.121774	4.498674	-0.056834
23	1	0	0.705080	5.447198	0.264116
24	6	0	2.375182	4.430242	-0.665877
25	1	0	2.962397	5.330374	-0.815842
26	6	0	2.854631	3.194548	-1.102050
27	1	0	3.811423	3.110640	-1.606225
28	6	0	2.066669	2.058496	-0.897730
29	7	0	1.995044	-0.419058	-0.638177
30	6	0	2.436396	0.715408	-1.481432
31	6	0	2.056242	-1.680172	-1.429064
32	1	0	2.975233	-1.722240	-2.029476
33	1	0	2.102458	-2.514464	-0.724698
34	6	0	2.820564	-0.520883	0.617557
35	1	0	2.352131	-1.307907	1.213403
36	1	0	2.683651	0.422396	1.153192
37	6	0	0.848923	-1.846013	-2.350159
38	1	0	1.024570	-2.712829	-3.006003
39	1	0	0.726919	-0.966152	-2.987852
40	6	0	-1.576512	-1.912178	-2.492817
41	1	0	-1.665506	-2.798039	-3.138701
42	1	0	-1.425595	-1.034498	-3.126986
43	6	0	-1.670088	-0.430082	3.271980
44	1	0	-1.518882	-1.509585	3.187787
45	1	0	-2.221944	-0.229739	4.200424
46	6	0	-0.269531	0.207388	3.356115
47	6	0	-2.191192	2.733328	-1.206000
48	1	0	-2.127897	3.821825	-1.318544
49	1	0	-3.225487	2.445375	-1.421619
50	6	0	-1.328659	2.046529	-2.281813
51	6	0	-0.428259	-3.303744	-0.854528
52	1	0	0.121858	-4.077834	-1.404587
53	1	0	-1.466910	-3.641155	-0.776003
54	6	0	0.098227	-3.217293	0.587661

55	7	0	-2.731545	-1.234940	-0.453643
56	7	0	-2.416088	0.027847	2.073083
57	7	0	-1.870002	2.313373	0.184395
58	7	0	0.883558	2.120711	-0.259287
59	7	0	-0.410750	-2.011654	-1.586001
60	8	0	0.180080	0.731816	2.258009
61	8	0	-0.940153	0.842549	-2.001481
62	8	0	0.012519	-2.052218	1.148982
63	1	0	1.918251	0.631946	-2.442710
64	1	0	3.513999	0.667220	-1.686940
65	6	0	4.298099	-0.807208	0.423451
66	6	0	5.226009	0.240686	0.298783
67	6	0	4.774202	-2.128672	0.386147
68	6	0	6.586588	-0.023072	0.114301
69	1	0	4.883071	1.270713	0.364050
70	6	0	6.133363	-2.396680	0.202412
71	1	0	4.078351	-2.953325	0.518794
72	6	0	7.042727	-1.343624	0.059960
73	1	0	7.288420	0.800604	0.021363
74	1	0	6.482354	-3.424964	0.179477
75	1	0	8.099592	-1.550538	-0.080183
76	8	0	0.518313	-4.251596	1.124260
77	8	0	-1.130012	2.643110	-3.348061
78	8	0	0.332976	0.149202	4.435913
79	65	0	-0.535247	-0.010174	0.143314

E(RTPSSh) = -2019.2220769 Hartree

Zero-point correction = 0.640725
Thermal correction to Energy = 0.679049
Thermal correction to Enthalpy = 0.679993
Thermal correction to Gibbs Free Energy = 0.570621
Sum of electronic and zero-point Energies = -2018.581352
Sum of electronic and thermal Energies = -2018.543028
Sum of electronic and thermal Enthalpies = -2018.542084
Sum of electronic and thermal Free Energies = -2018.651455
